# Identification and analysis of miRNAs in IR56 rice in response to BPH infestations of different virulence levels

**DOI:** 10.1038/s41598-020-76198-9

**Published:** 2020-11-05

**Authors:** Satyabrata Nanda, San-Yue Yuan, Feng-Xia Lai, Wei-Xia Wang, Qiang Fu, Pin-Jun Wan

**Affiliations:** State Key Laboratory of Rice Biology, China National Rice Research Institute, Hangzhou, 310006 China

**Keywords:** Entomology, Herbivory

## Abstract

Rice production and sustainability are challenged by its most dreadful pest, the brown planthopper (*Nilaparvata lugens* Stål, BPH). Therefore, the studies on rice-BPH interactions and their underlying mechanisms are of high interest. The rice ontogenetic defense, such as the role of microRNAs (miRNAs) has mostly been investigated against the pathogens, with only a few reports existing against the insect infestations. Thus, revealing the involvement of rice miRNAs in response to BPH infestations will be beneficial in understanding these complex interactions. In this study, the small RNA profiling of the IR56 rice in response to separate BPH infestations of varied virulence levels identified the BPH-responsive miRNAs and revealed the differential transcript abundance of several miRNAs during a compatible and incompatible rice-BPH interaction. The miRNA sequence analysis identified 218 known and 28 novel miRNAs distributed in 54 miRNA families. Additionally, 138 and 140 numbers of differentially expressed (DE) miRNAs were identified during the compatible and incompatible interaction, respectively. Gene Ontology (GO) and Kyoto Encyclopedia of Genes and Genomes (KEGG) enrichment analysis revealed the target gene candidates of DE miRNAs (including osa-miR2871a-3p, osa-miR172a, osa-miR166a-5p, osa-miR2120, and osa-miR1859) that might be involved in the IR56 rice defense responses against BPH infestation. Conversely, osa-miR530-5p, osa-miR812s, osa-miR2118g, osa-miR156l-5p, osa-miR435 and two of the novel miRNAs, including novel_16 and novel_52 might negatively modulate the IR56 rice defense. The expressional validation of the selected miRNAs and their targets further supported the IR56 rice defense regulatory network. Based on our results, we have proposed a conceptual model depicting the miRNA defense regulatory network in the IR56 rice against BPH infestation. The findings from the study add further insights into the molecular mechanisms of rice-BPH interactions and will be helpful for the future researches.

The normal human dietary uptakes depend largely on cereals, including rice (*Oryza sativa* L.), which is a staple food for more than half of the world population fulfilling more than 20% of the daily calorie needs^1^. It’s been estimated that by the year 2050, the global crop productions need to be increased at least 50% to satisfy the demand^2^. However, the current trend of rice production indicates only a 1.0% per year increase, which can be extrapolated to be a ~ 42% global increase by 2050, below par of the need^2^. In addition, rice productivity is severely challenged by several insect infestations^3^. Among them, the brown planthopper (*Nilaparvata lugens* Stål, BPH) is the most dreadful rice pest causing hopperburn, a fatal drying of rice plants that results in huge economic losses in Asia^1^. Although, the use of different insecticides is most common practice employed to control the BPH infestations, the abuse of these chemicals has resulted in many adversities, including insecticide resistance, insect resurgence, the elimination of natural enemies and other environmental hazards. Therefore, the identification and molecular breeding of rice germplasms continaing the BPH-resistance genes are considered to be the most suitable strategy for the control and management of BPH^1^. To date, 39 BPH resistance loci (*Bph*/*bph* genes) have been reported from different rice cultivars and from wild-rice species^4^. Amongst them, *bph2/Bph26*^5^, *Bph3/Bph17*^6^, *Bph6*^7^, *Bph9*^8^, *Bph14*^9^*, Bph15*^10^, *Bph18*^11^, *bph29*^12^, and *Bph32*^13^ have been isolated by map-based cloning. On one hand, the *Bph3* (a cluster of four plasma-membrane-localized lectin receptor kinases, *OsLecRK1-4*) has been considered for molecular breeding of rice cultivars with broad-spectrum and durable insect resistance^6,14^. On the other hand, it is of evident that these cultivars carrying the *Bph* genes can lose their resistance to BPH due to the evolution of new biotypes or populations^1,15,16^. Recently, a newly established virulent BPH population (IR56-BPH population) was discovered that could successfully break down the *Bph3*-mediated resistance in IR56 rice^16^. However, the underlying mechanisms for rice-BPH interactions is still unclear.

MicroRNAs (miRNAs) are of 21–24 nucleotides long endogenous regulatory non-coding small RNAs (sRNA) in plants having vital roles in the post-transcriptional regulation of gene expression during plant defense responses^17–19^. The early reports of their involvements in plant defense against herbivory were revealed from the sRNA transcriptome analysis of *Nicotiana attenuate*^20^. The silencing of RNA-directed RNA polymerase 1 (*RdR1*) and Dicer-like 3 (*Dcl3*) or Dicer-like 4 (*Dcl4*), important proteins from the miRNA biogenesis pathway, impaired the *N. attenuate* resistance against insect attacks. Further, the *RdR1* expression was induced either by application of jasmonic acid (JA) or salicylic acid (SA) or caterpillar oral secretions, but not by mechanical wounding itself. In *Cucumis melo*, the resistant Vat^+^ near isogenic lines and the susceptible Vat^−^ exhibited distinct miRNA profiles under the infestation of *Aphis gossypii*^21^. Likewise, in *Camellia sinensis* infestation of *Ectropis oblique* resulted in the differential expression of 150 miRNAs, supporting the role of miRNA in plant–insect interactions^22^. To our knowledge, the first study on the roles of miRNAs in rice-BPH interactions was reported in 2017, revealing the differential miRNA responses in a resistant (*BPH15* introgression line) and susceptible rice (recurrent parent 9311) in response to the infestation of BPH biotype 1^23^. However, to the best of our knowledge, no report exists on the role of miRNAs in a resistant rice variety in response to BPH infestations of variable virulence levels. Thus, the current scenario offers good opportunity to study the involvement of miRNA and their subsequent defense modulatory roles during the rice-BPH interactions.

In this study, the miRNA profiling of the resistant IR56 rice (carrying *Bph3*) have been performed under the independent infestations of a virulent IR56-BPH and an avirulent TN1-BPH. The IR56 rice and IR56-BPH interaction (hereafter referred as IR-*IR56-BPH*) is considered to be compatible, whereas the IR56 rice and TN1-BPH interaction (hereafter referred as IR-*TN1-BPH*) is considered to be incompatible in nature^24^. Small RNA sequencing data from three different libraries, i.e. IR-*IR56-BPH*, IR-*TN1-BPH*, and control (no BPH) were analyzed to find out the conserved and novel miRNAs involved in rice-BPH interactions. Further, the identification of the differentially expressed (DE) miRNAs in IR-*IR56-BPH* and IR-*TN1-BPH* suggested their roles in specific rice-BPH interactions. Additionally, the validation of some selected DE miRNA and their targets by qPCR analysis further strengthened their involvement in IR56 rice defense. The miRNA target predictions and their functional annotations by GO and KEGG enrichments indicated the defense modulatory roles of some DE miRNAs and their targets. Lastly, based on the findings of this study, a conceptual model depicting the miRNA defense regulatory network in IR56 rice has been proposed.

## Result

### Small RNAs sequencing in the IR56 rice

**Figure 1:**
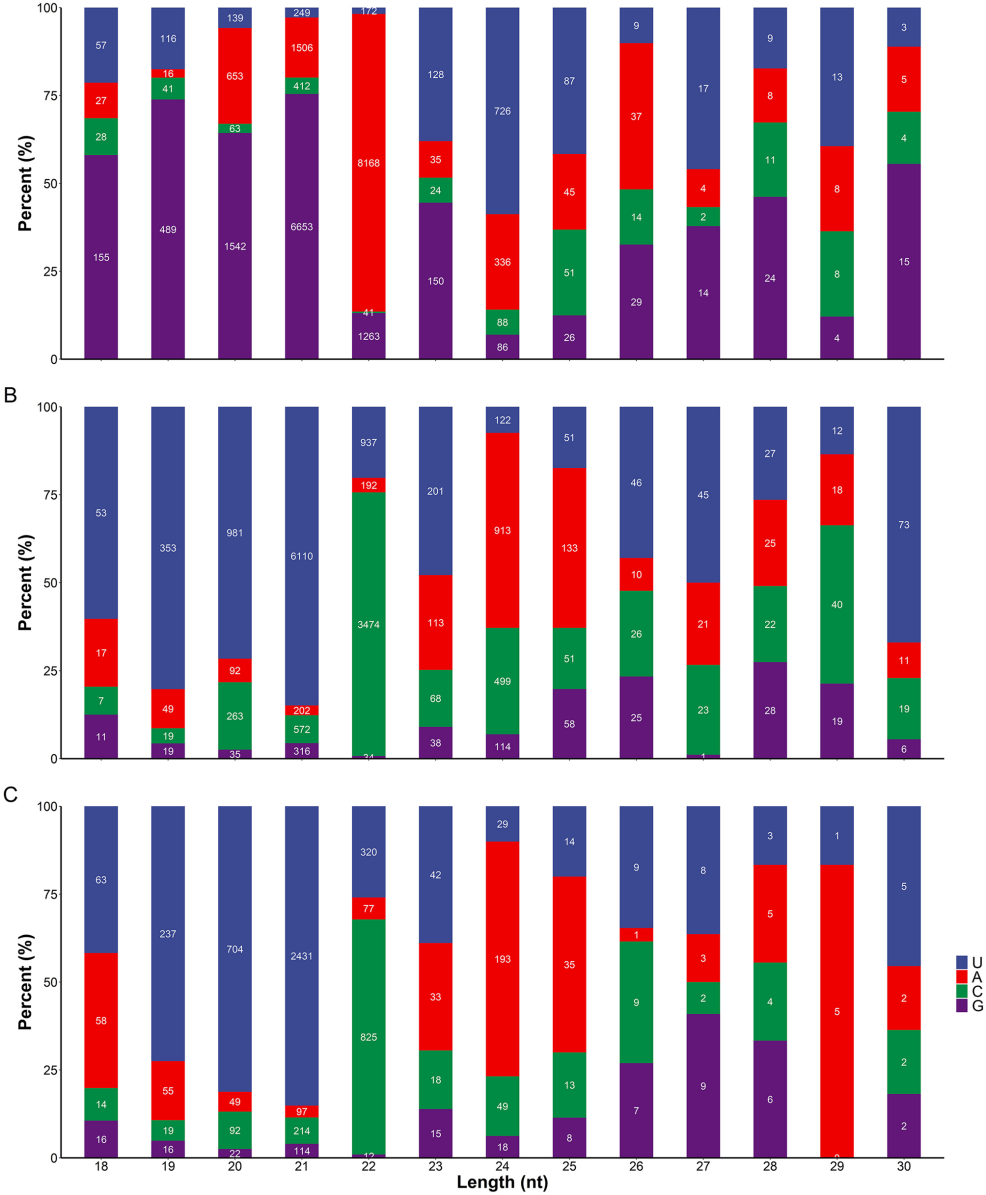
The first nucleotide bias (uridine, U; adenine, A; cytosine, C; guanine, G) at the 5′ end position of different lengths of the known miRNA in control (**A**), IR-*IR56-BPH* (**B**), and IR-*TN1-BPH* (**C**) libraries. The numbers of miRNA for each condition were denoted in the stacked histogram.

To reveal the involvement of miRNAs during the rice-BPH interactions, the small RNA sequencing was performed from the IR-*IR56-BPH*, IR-*TN1-BPH*, and control after 24 h of BPH feedings. Total raw reads of control, IR-*IR56-BPH*, and IR-*TN1-BPH* were 15296583, 12601079, and 16575853, respectively (Table 1). Raw reads of the three libraries were filtered to remove low quality reads, poly A, incorrect adaptors and sequences shorter than 18 nt. After sequence filtering, 14761791 (control), 12259165 (IR-*IR56-BPH*), and 15644891 (IR-*TN1-BPH*) clean reads were obtained (Supplementary Figure S1, Table 1). Subsequently, the alignment of all clean sequences with sRNAs in GenBank and Rfam databases resulted in the removal of sequences other than the unique reads. The obtained unique sequences were then mapped to the rice genome to exclude any matches with the exons or introns and repeat sequences. The remaining unique sequences were then aligned with the miRNA database in miRBase (release 21) to find out the known miRNAs. In total, these clean reads were mapped to 218 known miRNAs. The known miRNA length distributions of all three libraries were found to be mostly concentrated at 21 and 24 nt as reported in several plant species, including rice. Additionally, most of the 21 nt long miRNAs contained the 5′U as the first base (Fig. 1).

**Table 1:** The summary of sRNA sequencing result data.

Sequence type	Control			IR56-BPH			TN1-BPH		
	Raw counts	Unique counts	Total counts	Raw counts	Unique counts	Total counts	Raw counts	Unique counts	Total counts
Raw reads	9,128,932	417,277	9,546,209	7,400,347	439,295	7,839,642	8,812,250	336,072	9,148,322
Clean reads	3,032,174	159,462	3,191,636	2,325,929	175,454	2,501,383	2,763,080	134,601	2,897,681
rRNA	8,520,462	160,027	8,680,489	6,720,834	165,447	6,886,281	8,448,361	157,756	8,606,117
tRNA	26,782	2566	29,348	39,360	3136	42,496	25,390	2412	27,802
snRNA	2853	1054	3907	3809	1250	5059	2146	911	3057
snoRNA	17,043	3966	21,009	21,350	4378	25,728	11,999	3394	15,393
Repeats	205,796	94,997	300,793	202,490	100,147	302,637	47,939	29,365	77,304
Exons	164,544	76,430	240,974	167,697	79,725	247,422	126,311	73,424	199,735
Introns	19,312	9384	28,696	23,323	11,159	34,482	11,660	6766	18,426
Others	172,140	68,853	240,993	221,484	74,053	295,537	138,444	62,044	200,488

### Identification and expression of known and novel miRNAs

A total of 218 known rice miRNAs belonging to 54 rice miRNA families, and 28 novel miRNAs were discovered in the three libraries (Supplementary Table S1). Among all identified known and novel miRNAs, six miRNAs were detected only in IR-*TN1-BPH*, 24 miRNAs were found only in IR-*IR56-BPH*, and 31 miRNAs were only found in the control (Supplementary Table S2), while 185 remaining miRNAs were found in all three libraries.

The miRNAs having more than 1000 transcripts per million (TPM) were considered as abundantly expressed miRNAs, while those with less than 10 TPM were classified as rarely expressed miRNAs. The 20 most abundant miRNAs in each of the libraries (accounting for ~ 90% of total miRNA reads) are listed in Table 2. Two of them (novel_117, and novel_121, shown in bold) were novel miRNAs. The number of rare miRNAs in TN1-BPH (98) was twice the number of IR56-BPH (43), while showed no significant difference between IR56-BPH (43) and control (35), indicating that many miRNAs may be down-regulated in incompatible rice-BPH interaction (Supplementary Table S3).

**Table 2:** Top 20 most abundant miRNAs expressed in the four libraries (TPM were shown).

miRNA	IR56_CK	miRNA	IR56_IR	miRNA	IR56_TN
osa-iR1861b	395,432.94	osa-miR1861b	249,283.49	osa-miR1861b	173,834.54
osa-iR396f.-5p	62,370.48	osa-miR396f.-5p	97,201.05	osa-miR396f.-5p	121,963.23
osa-iR396e-5p	62,207.96	osa-miR396e-5p	96,835.17	osa-miR396e-5p	121,963.23
osa-miR166a-3p	57,657.15	osa-miR166a-3p	78,968.23	osa-miR166a-3p	106,040.71
osa-miR166k-3p	41,891.84	osa-miR162a	46,466.25	osa-miR159a.1	43,663.82
osa-miR167d-5p	35,756.37	osa-miR167d-5p	36,099.76	osa-miR166k-3p	40,052.53
**novel_121**	32,830.85	osa-miR166k-3p	35,977.80	osa-miR166g-3p	39,067.63
osa-miR1861a	30,311.65	osa-miR159a.1	35,977.80	osa-miR167d-5p	36,277.08
osa-miR162a	27,508.02	osa-miR1862e	30,245.75	osa-miR162a	24,130.01
osa-miR159a.1	23,607.33	osa-miR166g-3p	25,428.38	osa-miR1861a	17,564.02
osa-miR1861h	17,959.45	osa-miR812k	23,355.08	osa-miR156a	14,937.62
osa-miR166g-3p	17,918.82	osa-miR1861a	19,147.51	osa-miR1862d	14,773.47
osa-miR1862e	14,018.12	osa-miR1862d	18,354.78	osa-miR166j-5p	14,445.17
osa-miR1862d	11,864.61	osa-miR166j-5p	14,513.08	osa-miR396g	13,788.58
osa-miR812k	11,458.29	osa-miR396c-5p	8110.25	osa-miR166m	12,967.83
osa-miR166j-5p	10,523.75	osa-miR166m	7500.46	osa-miR812k	11,654.63
**novel_117**	8085.82	osa-miR5794	7195.56	osa-miR168a-5p	8699.93
osa-miR396c-5p	8004.55	osa-miR168a-5p	6829.68	osa-miR396c-5p	8207.49
osa-miR166m	6907.48	osa-miR820a	6768.71	osa-miR11337-5p	7222.59
osa-miR399d	6541.79	osa-miR156a	5671.08	osa-miR1862e	7058.44

### Identification of DE miRNAs in response to BPH infestations

**Figure 2:**
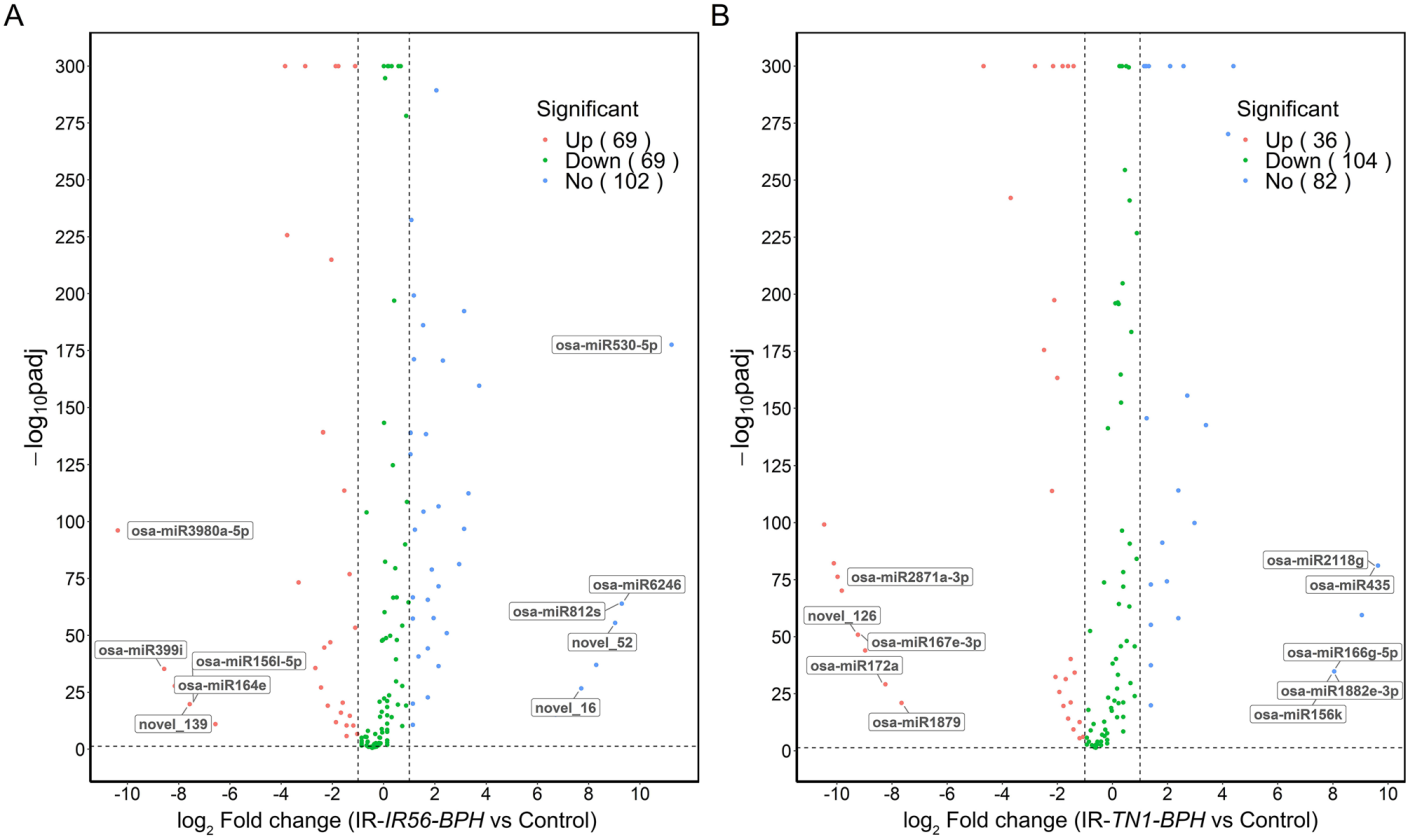
The volcano plots showing the DE miRNAs in the comparisons of IR-*IR56-BPH* versus control (**A**) and IR-*TN1-BPH* versus control (**B**). The significantly upregulated, and downregulated miRNAs were shown in red and green, respectively (adjusted *P* value < 0.01). No differential expression between the two groups was shown in blue (adjusted *P* value > 0.01). The number of genes in each group was parenthesized. The randomly selected 20 miRNAs in following qRT-PCR were labelled. The miRNAs that were not expressed in both libraries were not shown in the figure.

The differential expression and Pearson correlation analysis of the miRNAs revealed the DE miRNAs in IR56 rice in response to BPH infestations. The correlation analysis indicated a positive correlation among all the samples used in the sRNA sequence analysis (Supplementary Figure S2). The differential expression analysis of all identified miRNAs (known and novel) revealed that BPH infestations have significant effects on the transcript abundance of the IR56 rice miRNAs. Out of the 246 identified miRNAs, 138 for IR-*IR56-BPH* and 140 for IR-*TN1-BPH* were found to be deferentially expressed, compared with the control (no BPH) (Supplementary Table S3). In the IR-*IR56-BPH*, 69 numbers of miRNAs were found to be upregulated, whereas 69 miRNAs were down regulated in comparison to the control (Fig. 2A). On the other hand, 36 miRNAs were found to be upregulated in the IR-*TN1-BPH*, while 104 miRNAs were downregulated (Fig. 2B). Additionally, some DE miRNA families were found to be exclusive in the IR-*IR56-BPH* or IR-*TN1-BPH*, respectively. For instance, miRNAs from the families like MIR397 and MIR398 were found exclusive to the IR-*IR56-BPH* but not in IR-*TN1-BPH*. Conversely, miRNAs from the families like MIR172 and MIR435 were found exclusively in the IR-*TN1-BPH* but not in IR-*IR56-BPH*. Collectively, the DE miRNA analysis results in the IR-*IR56-BPH* and IR-*TN1-BPH* libraries suggested that different miRNAs are involved in the rice-BPH interactions and the kind of interaction (compatible or incompatible) affects the number (how many), kind (which family/novel), and nature (up or downregulation) of the miRNA expressions in rice.

### Prediction of the targets of the DE miRNAs and their functional annotations

**Figure 3:**
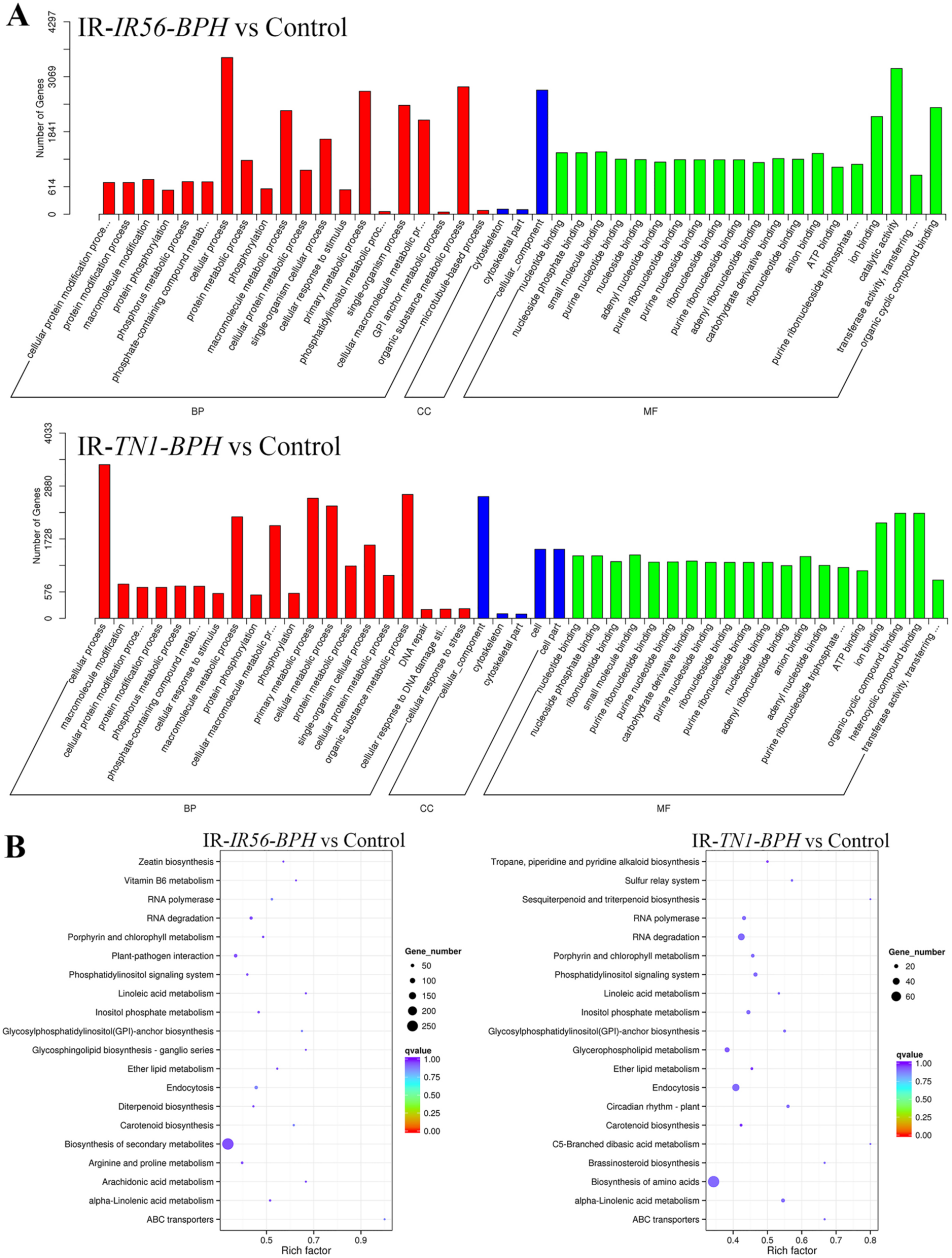
Gene ontology annotations (**A**) and top 20 KEGG pathways (**B**) enriched in the DE miRNA targets in IR-*IR56-BPH* and IR-*TN1-BPH*.

The prediction of the targets of the DE miRNAs provided a means to understand the possible defense modulatory roles of the miRNAs. Also, the analysis revealed that a single miRNA can have multiple targets in rice, whereas a single mRNA transcript can also be targeted by multiple miRNAs. To functionally characterize the miRNA targets, GO and KEGG enrichments of the predicted targets were carried out. The GO term annotations revealed similar biological process and molecular functions for the targets in both IR-*IR56-BPH* and IR-*TN1-BPH*, such as metabolic process and protein modifications, and nucleotide-binding and transferase activity (Fig. 3A). However, the targets associated with IR-*TN1-BPH* DE miRNAs have crucial stress-responsive GO enrichments indicating their possible roles in rice defense responses. Similarly, the KEGG pathway enrichments revealed that some of the target genes of the miRNAs from IR-*IR56-BPH* and IR-*TN1-BPH* samples are commonly involved in the physiological processes, including RNA degradation, glycosylphosphatidylinositol (GPI)-anchor biosynthesis, lipid metabolisms, carotenoid biosynthesis inositol metabolism and phosphatidylinositol signaling, and endocytosis (Fig. 3B). However, the IR-*IR56-BPH* and IR-*TN1-BPH* targets had some different and exclusive KEGG enrichments such as, zeatin biosynthesis, plant-pathogen interaction, and vitamin B6 metabolism for the IR-*IR56-BPH*, while brassinosteroid biosynthesis, circadian rhythm, and sulfur relay system for the IR-*TN1-BPH*. Thus, having the common metabolic pathway enriched targets in IR-*IR56-BPH* and IR-*TN1-BPH* suggest that the BPH-feeding response in IR56 rice share several common features immaterial with the type of BPH population, whereas exclusive pathway enrichments to IR-*IR56-BPH* or IR-*TN1-BPH* miRNA targets suggest the differential metabolic response in IR56 rice to BPH feedings depending on the infested population.

### Expression validation of selected DE miRNAs and their targets

**Figure 4:**
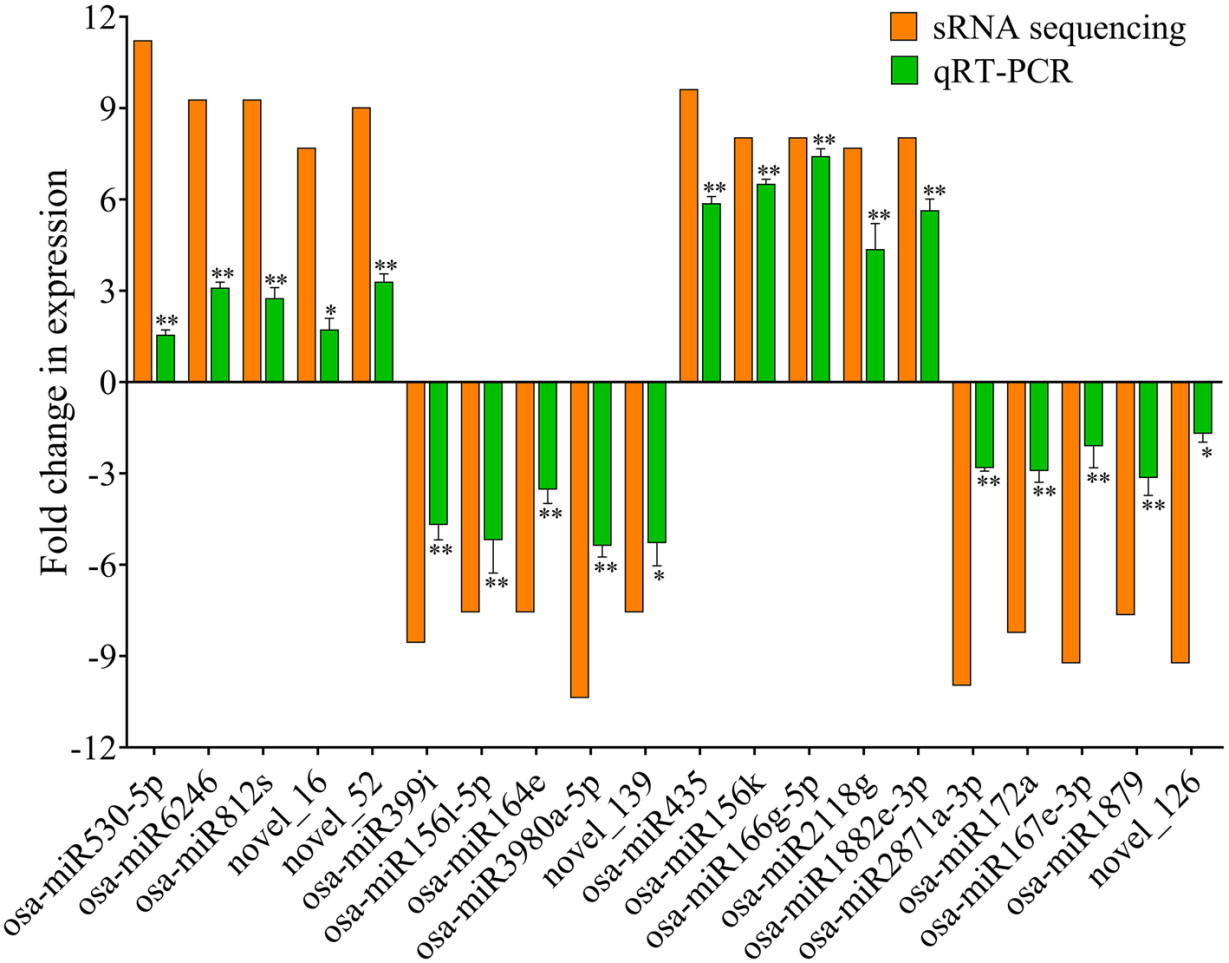
Expression validation of the selected miRNAs by qPCR. The fold changes (log_2_) in the expression of the miRNAs were calculated and compared to the sRNA sequencing data. Bars represent the mean ± SE of three biological replicates for the qPCR data. Asterisks * and ** indicate the significant difference in the expression levels of miRNAs in IR-*IR56-BPH* or IR-*TN1-BPH* as compared to control at *P* < 0.05 and *P* < 0.01, respectively (Student’s *t*‐test).

To validate the transcript abundance of the identified miRNAs in the sRNA sequencing, we have randomly selected 20 miRNAs (10 each from IR-*IR56-BPH* and IR-*TN1-BPH*) and their relative expressions were evaluated by real-time quantitative PCR (qPCR, primers listed in Supplementary Table S4). The qPCR results were found to be in accordance with the sRNA sequencing results indicating a similar and significant trend of relative expression levels in the BPH infested rice samples as compared to the control (Fig. 4). However, the degree of the relative fold changes of the DE miRNAs obtained via sRNA sequencing and qPCR analysis did differ. Thus, the similar expression profiles of the miRNAs between the qPCR and sRNA sequencing indicated the sequencing results to be reliable and suitable for further analyses.

**Figure 5:**
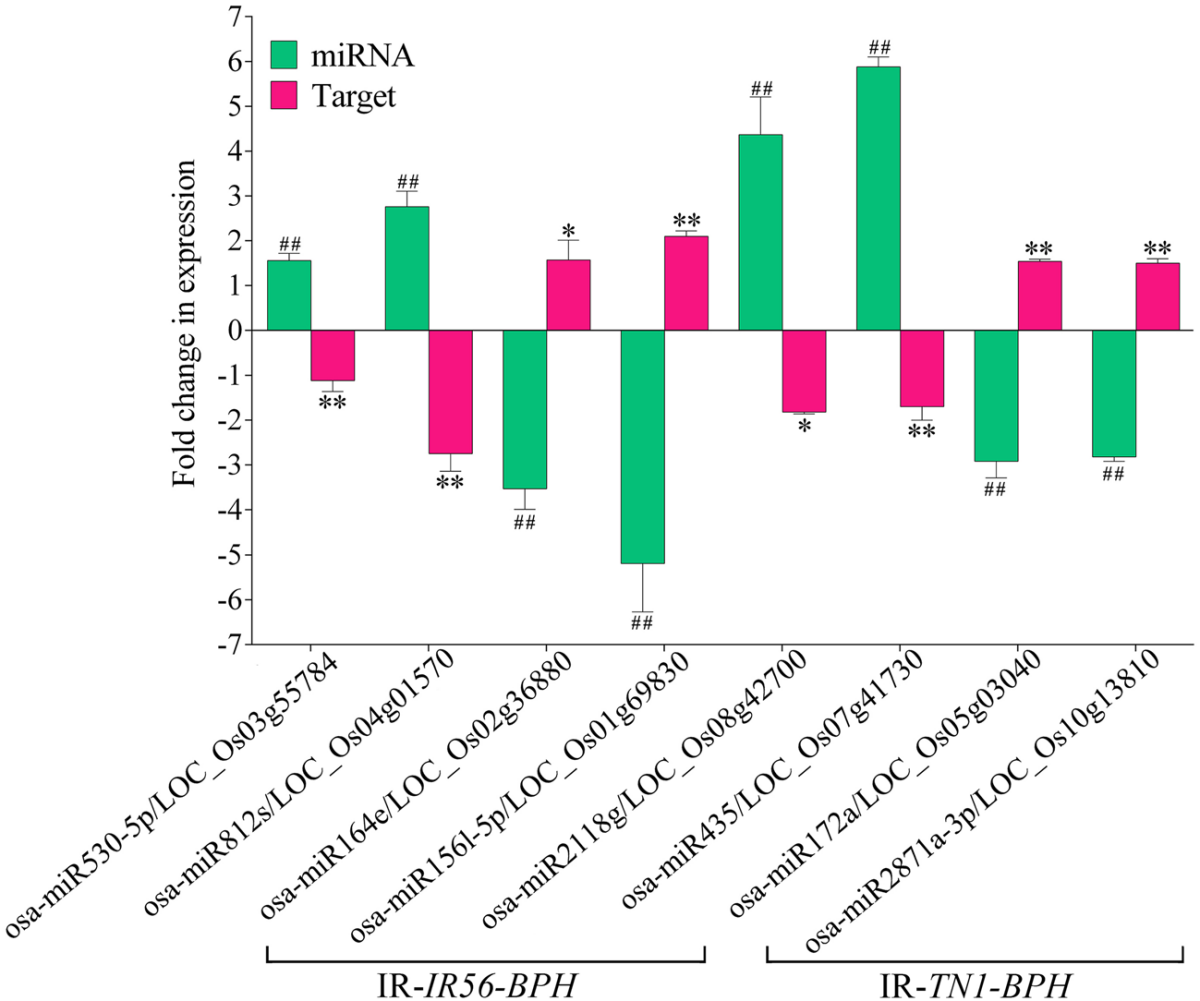
Expression validation of the selected miRNAs targets by qPCR. The fold changes (log2) in the expression of the target genes were calculated and compared to those of the miRNAs in IR56 rice. Bars represent the mean ± SE of three biological replicates for the qPCR data. Hashtags # and ## represent the significant difference in the expression levels of miRNAs and asterisks * and ** indicate the significant difference in the expression levels of target genes in IR-*IR56-BPH* or IR-*TN1-BPH* as compared to control at *P* < 0.05 and *P* < 0.01, respectively.

The target gene predictions of the DE miRNAs have resulted in identifying several genes in the IR56 rice associated with defense responses and plant protection (Supplementary Table S5). Additionally, the GO and KEGG enrichments further strengthen their roles in rice defense. From the predicted targets, we selected 8 genes and validated their relative expressions by qPCR. All the selected targets exhibited a negative correlation to their corresponding miRNA expression levels (Fig. 5). In the IR-*IR56-BPH*, strong downregulation (log_2_ fold change > 2) was observed in the target of osa-miR812s (*LOC_Os04g01570*) that encodes a pectin methylesterase inhibitor (PEMI) protein. Significant downregulation in its transcript accumulation in IR-*IR56-BPH* was also observed in the target of osa-miR530-5p (*LOC_Os03g55784*) that encodes an allene oxide synthase (AOS). Conversely, the targets of the downregulated miRNAs in IR-*IR56-BPH*, including osa-miR3980a-5p and osa-miR156l-5p were found to show significant upregulated expression levels as compared to the control. These targets include *LOC_Os01g69830* and *LOC_Os02g36880*, encoding a squamosa promoter binding protein (SBP) and a no apical meristem (NAM) protein, respectively. In the IR-*TN1-BPH*, the targets of the upregulated miRNAs osa-miR2118g and osa-miR435 were found to be significantly downregulated, including *LOC_Os08g42700* (encoding a NB-ARC domain containing protein) and *LOC_Os07g41730* (encoding an alpha/beta hydrolase domain containing protein), respectively. On the other hand, the targets of the downregulated miRNAs osa-miR2871a-3p and osa-miR172a in IR- *TN1-BPH* were found to show significant upregulations, including *LOC_Os10g13810* (encodes a glycosyltransferase family protein) and *LOC_Os05g03040* (encodes an AP2/EREBP family transcription factor), respectively. The negative correlations in between the DE miRNAs and their targets suggest the existence of a well-orchestrated post-transcriptional regulation in response to the BPH infestation in rice. Moreover, the transcript dynamics of different target genes depending upon the nature of rice BPH interactions (IR-*IR56-BPH* or IR-*TN1-BPH*) indicate the possible existence of a differential repertoire of defense-related transcription factors/proteins for the compatible or incompatible interactions.

## Discussion

Extensive researches on understanding the roles of miRNAs in plant physiology and protection have resulted in the discovery and characterization of more numbers of miRNAs than ever before. Although, many works depicting the role of miRNAs in the plant-aphid interactions have been reported^25^, only a few reports indicating the role of rice miRNA in insect defense response is available^23,26,27^. To the best of our knowledge, this is the first report briefing the miRNA dynamics in a resistant rice in response to the infestations of a virulent and an avirulent BPH population. In the current work, the comparative sRNA sequencing of IR56 rice infested separately by the virulent IR56-BPH (IR-*IR56-BPH*, compatible interaction) and the avirulent TN1-BPH (IR-*TN1-BPH*, incompatible interaction) revealed the differential involvement of miRNAs during BPH feeding. Besides, comparisons among the sRNA libraries of the BPH-infested and the control (no BPH) revealed that BPH feeding reprogrammed the miRNA expressions in the IR56 rice. A total of 278 DE miRNAs were found in response to the BPH infestations, out of which we have identified 28 mature novel and 218 mature conserved miRNAs. Further, the exclusive nature of the presence of some miRNA families in either IR-*IR56-BPH* or IR-*TN1-BPH* samples indicated the interaction-specific nature of those miRNA families in IR56 rice. The exclusive nature of tissue-specific expression of several miRNA families has been reported in a rice cultivar in response to drought stress^28^. In addition, several miRNA families showed exclusive differential expressions during the interactions between two rice cultivars (*Bph15* IL and 9311) and BPH biotype 1^23^. On the contrary, 71 DE miRNAs (18 up and 53 downregulated) were found to be in common in between IR-*IR56-BPH* and IR-*TN1-BPH* suggesting that several miRNAs act in a similar pattern in response to BPH feeding irrespective of their virulence levels. The relative transcript abundances of some of the selected DE miRNAs were validated by qPCR analysis, which further strengthened the reliability of the sRNA sequencing data.

In response to the IR56-BPH infestation, osa-miR530-5p and osa-miR812s were found to be highly upregulated in the IR-*IR56-BPH* samples. The *MIR530* family members, including osa-miR530-5p have been reported to be involved in stress responses^29–31^. The target prediction results revealed that the osa-miR530-5p target *LOC_Os03g55784* encodes an AOS that participate in the JA signaling in rice^32^. JA has been reported to be associated with insect defense responses in rice, and also might act as an early event in rice-BPH interaction^24,33^. Thus, the elevated transcript abundance of osa-miR530-5p in the IR-*IR56-BPH* during the feedings of the virulent IR56-BPH might have tampered the JA signaling pathway, a common signaling event to insect attacks in rice. Manipulation of JA signaling in rice in response to BPH infestation has also been reported to be achieved by other miRNA family members, including miR160f-3p, miR166c-5p and miR169r-3p^23^. In addition to this, the target of osa-miR812s, *LOC_Os04g01570*, encodes a PEMI protein reported to be involved in plant growth and stress responses^34^. Although, PEMIs have been reported to play significant roles in the growth and development in rice^35,36^, several recent reports have revealed their importance in biotic stress tolerance in plants, including *Arabidopsis* and cotton^37,38^. Thus, the upregulated expression of osa-miR812s during the compatible rice-BPH interaction, resulting in the downregulation of PEMI proteins in the IR56 rice might have aided in the successful rice resistance breakdown. In addition, in the IR-*IR56-BPH* plants the elevated transcript abundances of two novel miRNAs, novel_16 and novel_52, were observed that target a serine/threonine (S/T) kinase and a lectin kinase protein, respectively. The roles of S/T kinases and lectin kinases in rice resistance response have already been discovered^10,39^. Further, the *OsLecRK1-4* have been identified to confer broad-spectrum resistance in rice^6^. Our previous study revealed that in IR56 rice *OsLecRK3* and *OsLecRK4* displayed induced expressions during the incompatible rice-BPH interactions^24^. Thus, the downregulation of the S/T kinase or the lectin kinases, possibly by the miRNA-mediated transcript cleavage might have favored a continuous feeding and resistance breakdown in the IR-*IR56-BPH* plants. Conversely, downregulations of several miRNAs were also observed during the compatible IR-*IR56-BPH* interactions, including the osa-miR156l-5p. The osa-miR156 is a conserved miRNA having a usual target of the SPB transcription factors^40,41^. Target mimic-based silencing of the miR156 that targets multiple SBP proteins in rice has resulted in the enhanced resistance response against BPH infestation^26^. Further, downregulation of *OsSPL2* by the overexpression of osa-miR529 had conferred enhanced stress resistance in rice by the elevated expression of *superoxide dismutase* (*SOD*) and *peroxidase* (*POD*) genes^42^. However, the lower transcript abundance of osa-miR156l-5p in the IR-*IR56-BPH* plants resulted in the upregulated expression of *OsSPL2* (LOC_Os01g69830), which might have suppressed the POD and SOD transcription, thereby decreasing the reactive oxygen species (ROS)-signaling events. ROS-signaling might act as a basal defense response and an early event in rice-BPH interactions^24,43^. Thus, these consequences of the upregulated expression of osa-miR156l-5p might have helped IR56-BPH to overcome the rice resistance.

**Figure 6:**
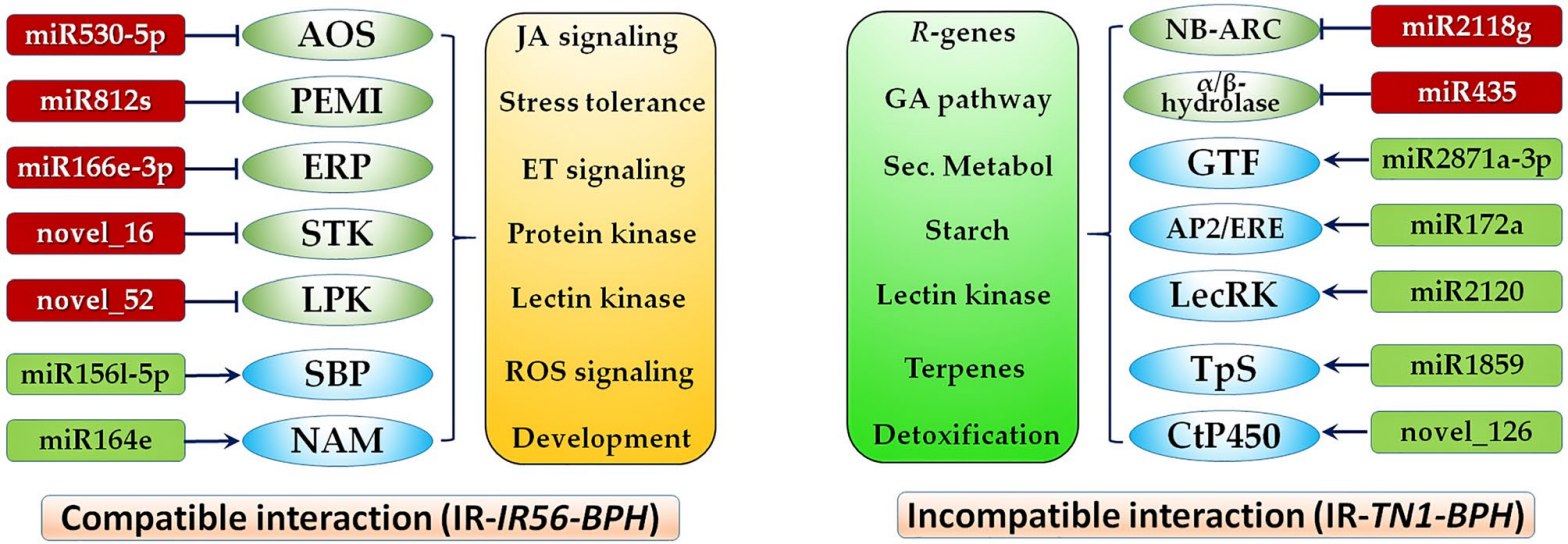
A conceptual model depicting the miRNA regulatory network of the IR56 rice against the infestation of IR56-BPH and TN1-BPH. AOS: allene oxide synthase; PEMI: pectin methylesterase inhibitor; ERP: ethylene-responsive protein; STK: serene/threonine protein kinase; LPK: lectin protein kinase; SBP: sqamosa promoter-binding protein; NAM: no apical meristem; ET: ethylene; SA: salicylic acid; ETS: effector-triggered susceptibility; GA: gibberellins; NB-ARC: NB-ARC domain containing proteins; GTF: glycosyltransferase; AP2/ERE: AP2/ERE domain containing transcription factor; LecRK: lectin receptor kinase; CtP450: cytochrome P450; Sec.Metabol: secondary metabolites. Arrows indicate a positive correlation, whereas blunt ended lines represent a negative correlation.

In the TN1-BPH infested IR56 rice (IR-*TN1-BPH*), the elevated transcript accumulations of osa-miR2118g and osa-miR435 were observed and verified by qPCR. *LOC_Os08g42700*, encoding a NB-ARC domain containing protein was predicted as a target of osa-miR2118g, whereas *LOC_Os07g41730*, encoding an α/β-hydrolase was predicted to be the target of osa-miR435. The α/β-hydrolase has been reported to serve as the core structure for phytohormones and ligand receptors, including that of gibberellins (GA)^44^. GA has been reported to positively regulate rice defense against BPH infestations, as the overexpression of GA receptor *OsGID1* enhanced BPH resistance in rice^45^. But the downregulation of α/β-hydrolase that is associated with the GA pathway might be induced by the TN1-BPH feedings as an attempt to disrupt rice defenses. Further, the NB-ARC or NB-LLR proteins have been extensively studied in plants, including rice, specifically for their roles in defense responses^46^. Also, the phytopathogen-induced miRNA-mediated suppression of rice defense genes has been reported during the rice blast disease^47^. Thus, the suppression of the NB-ARC protein in rice mediated by the upregulated expression of osa-miR2118g could be an attempt by the TN1-BPH to outrun the IR56 rice defense. Ouyang and colleagues proposed that due to the transcript abundance of miRNAs that targets the NB-domain genes in tomato, the susceptible cultivars express insufficient resistance proteins^48^. Besides, as the NB-ARC family forms a vital class of *R*-genes, interacting with the pathogen/insect effectors, osa-miR2118g might participate in the ETI response to channel the defense response to BPH. On the other hand, more numbers of miRNAs exhibited a reduced transcript abundance in the IR-*TN1-BPH* plants. For instance, osa-miR2871a-3p, osa-miR172a, osa-miR166a-5p, osa-miR2120, and osa-miR1859 were found to be downregulated many folds as compared to the control. A glycosyltransferase family protein (*LOC_Os10g13810*) was predicted to be the target of osa-miR2871a-3p has been associated with multiple functions in rice, including growth, development, and stress responses^49,50^. Two glycosyltransferase genes *UGT73B3* and *UGT73B5* were identified to be necessary for the pathogen defense in *Arabidopsis*^51^. In addition, UDP-glycosyltransferase was reported to facilitate the modifications and storage of secondary metabolites in rice and thereby defending against stress^50^. Thus, the transcript accumulation of *LOC_Os10g13810* in IR-*TN1-BPH* might have boosted the rice resistance, possibly by regulating the secondary metabolite pool. Dai et al. has reported that by sequestering osa-miR396 the rice defense response against BPH can be enhanced via the increased biosynthesis of flavonoids^27^. Our previous findings also supported the positive role of secondary metabolites, such as phenylpropanoids in the rice-BPH interactions^24^. Additionally, upregulation of *LOC_Os01g23530* (the predicted target of osa-miR1859) encoding a terpene synthase further supports this hypothesis. Another gene *LOC_Os05g03040* (target of osa-miR172a), encoding an AP2/EREBP family transcription factor and participating in starch biosynthesis was found to exhibit upregulated expressions in IR-*TN1-BPH* plants. It is evident that during a compatible rice-BPH interaction, rapid starch breakdown occurs to produce more sucrose in rice plants as a large amount of sucrose is consumed by the BPH feeding^1^. On the contrary, during the incompatible rice-BPH interaction the resistant rice plants produce more starch indicating lesser loss of sucrose, showing resistance to the BPH feedings^1,52^. Thus, our results from this study are in accordance with the previous report, as in the IR-*TN1-BPH* plants the starch biosynthesis is upregulated, supporting the IR56 rice resistance to the TN1-BPH. In addition to this, downregulation of osa-miR2120 and thus, the upregulation of its target *LOC_Os02g32680*, encoding a lectin receptor-type protein kinase was observed in the IR-*TN1-BPH* plants. The upregulation of the lectin receptor kinase are believed to be crucial for the rice defense responses against BPH infestation during an incompatible interactions^6,24^. Thus, these results further support the incompatible nature of IR56 rice and TN1-BPH interactions and the vital regulatory roles of miRNAs. On the basis of the miRNA results obtained in this study, their target prediction and functional annotations, and the validation of some miRNA and target gene negative correlations, a conceptual model depicting the miRNA regulatory network of the IR56 rice in response to BPH feedings from two different populations of varied virulence levels have been proposed (Fig. 6). The findings from this study added new information about the involvement of miRNAs and their defense modulatory roles in response to BPH feedings on the resistance IR56 rice.

## Conclusion

In conclusion, the comparative miRNA profiling of the IR56 rice infested separately by the virulent IR56-BPH and the avirulent TN1-BPH revealed the dynamics of miRNA pool. Identification of the DE miRNA indicated that BPH feeding reprogramed the miRNA transcriptions in the IR56 rice. Although, the total numbers of DE miRNAs were found to be similar in IR-*IR56-BPH* and IR-TN1-BPH, but more numbers of miRNAs were found to be downregulated during the incompatible interactions, suggesting the defense modulatory roles of their targets. Prediction of targets and their functional enrichments by GO and KEGG analysis added more insights to their putative functionality in rice-BPH interactions. Validation of the expression profiles of selected miRNAs and their predicted targets further strengthened their involvements in rice defense against BPH attacks. Finally, based on the findings of this study, a conceptual model depicting the regulatory network of IR56 rice in response to the BPH infestations has been proposed. Furthermore, to the best of our knowledge, this is the first report on the differential rice miRNA roles in a resistance rice in response to BPH infestations of different virulence level. In depth analysis experiments, such as overexpression or selective mutation of a miRNA or the miRNA target MIMIC studies will further validate the functional roles in rice resistance of the identified candidate miRNA in this study. Moreover, the findings from this study will add new insights to the defense regulatory roles of rice miRNA in response to BPH attacks and will help to understand the rice-BPH interactions.

## Materials and methods

### Ethics statement

All animal work has been conducted according to the relevant national and international guidelines.

### Plant material and growth

An *indica* rice variety ‘IR56’ was used in this experiment as the plant material. In a net house, pre-germinated IR56 rice seeds were planted in mud beds and grown under natural light and temperature conditions. After 14 days old seedlings were then transplanted into mud-filled cups (diameter 12 cm, height 15 cm) and put in the China National Rice Research Institute (CNRRI) greenhouse with following growth conditions: temperature was set to 28 ± 2 °C with an 80 ± 5% relative humidity RH. Ten days post transplantation, the IR56 rice plants were used for conducting the experiments.

### Insect materials

Two BPH populations, i.e. TN1-BPH and IR56-BPH of different virulence were used to perform the BPH bioassays in this study^24^. BPH colonies initially collected from rice fields in Hangzhou, China, were maintained on Taichung Native 1 (TN1) rice (TN1-BPH) or IR56 rice (IR56-BPH) in a climate-controlled chamber (26 ± 2 °C, 80 ± 5% RH) for more than 7 years at the CNRRI^24^.

### BPH bioassays and sample collections

Individual IR56 rice plants were infested with 4 newly emerged adult females BPH, and were confined in a transparent plastic cage (diameter 10 cm, height 60 cm) equipped with a net (with holes of diameter 0.5 mm)^24^. As per the aforementioned procedures, both TN1-BPH and IR56-BPH populations were infested onto the separate IR56 rice plants. The interactions in between IR56-BPH with IR56 rice (IR-*IR56-BPH*) was considered as the compatible, whereas the interaction in between TN1-BPH and IR56 rice (IR-*TN1-BPH*) was considered to be the incompatible rice-BPH interaction^24^. IR56 rice plants with no BPH treatment put inside a plastic cage served as a control for this experiment. The BPH assay experiments were performed with 3 independent biological replicates. After 1 day of the BPH infestations, the stem portions of the plants (control and treated) were collected and immediately frozen in liquid nitrogen. The replicates of each sample were combined as one and stored at − 80 °C until further use.

### Small RNA library construction and sequencing

Total RNA was isolated from each sample (Control, no BPH; IR56-BPH infested, IR-*IR56-BPH*; TN1-BPH infested, IR-*TN1-BPH*) by using the TransZol Up reagent (Transgen, Beijing, China) according to the manufacturer’s instructions. The purity, concentration and integrity of isolated RNA were determined by a NanoPhotometer spectrophotometer (IMPLEN, CA, USA), Qubit RNA Assay Kit in Qubit 2.0 Flurometer (Life Technologies, CA, USA), and Agilent 2100 (Agilent Technologies, CA, USA), respectively. The RNA degradation and contamination was monitored on 1% (w/v) agarose gel electrophoresis. Total RNA (> 3 μg) of good quality was used to construct an sRNA library for each sample by using NEBNext Multiplex Small RNA Library Prep Set for Illumina (NEB, USA.) following manufacturer’s recommendations. To the enriched small RNA pool, 5′ and 3′ adapters were ligated by T_4_ RNA ligase. Complementary first strand cDNAs were PCR amplified to generate cDNA libraries. Subsequently, the libraries were sequenced (single-end) on an Illumina Hiseq2500 at the Novegene Company (Beijing, China) following the vendor’s recommended protocol. The sequencing data have been submitted to the NCBI’s GEO database.

### Data analysis and identification of the differentially expressed miRNAs

After Illumina sequencing, raw data were processed using Novogene’s Perl and Python scripts. Clean data were screened to remove reads containing more than three N (undetermined bases), reads with 5′ adapter contaminants, reads without 3′ adapter or the insert tags, those containing poly A, T, G, or C and low quality reads obtained from the raw data. Then, sRNA sequences of 18–35 nt were selected to conduct all downstream analyses. To prevent every unique sRNA mapping to multiple non-coding RNA (ncRNAs), we used the following priority rule: known miRNA > rRNA > tRNA > snRNA > snoRNA > repeat > gene > novel miRNA so that every unique sRNA mapped to only one annotation. The Bowtie v1.2.3^53^ was used to map the sRNA tags to the *indica* rice ShuHui498 (R498) genome (https://www.mbkbase.org/R498/)^54^ without mismatch to analyze their expression and distribution on the reference sequence. Next, the mappable sRNA tags were aligned to the miRNA precursor of *O. sativa* in the miRNA database (miRbase v. 22.1) to obtain the known miRNA count. Then, rRNAs, tRNAs, snRNAs, and snoRNAs were removed by mapping the remained sRNA tags to Rfam release 14^55^. Repeat sequences were filtered by using a repeat sequences database^56^, and tags originating from protein coding genes were discarded by mapping to the exon and intron of mRNAs of *O. sativa*. Finally, novel miRNAs were predicted by exploring the secondary structure, the Dicer cleavage site and the minimum free energy of the former unannotated sRNA tags which could be mapped to the reference sequence by integrating two available software miREvo^57^ and mirdeep2^58^.

Since the biological replicates of each samples were combined as one bulk, therefore, when analyzing differentially expressed miRNAs between libraries, we first transformed the raw read count matrix of miRNAs into TPM (transcript per million)^59^, then used the DEGseq R package^60^ to analyze the differences. The adjusted *P* value (q Value) < 0.01 and absolute value of log_2_ (fold change) > 1 was set as the threshold for significant differential expression by default^61^. We compared the expression level of miRNAs between IR-*IR56-BPH* versus control, and IR-*TN1-BPH* versus control.

### Prediction and functional annotations of the miRNA targets

The targets of the identified DE miRNAs in the *O. sativa* genome were predicted using the TargetFinder software^62^. To further reveal functions related to the putative target genes, GO (https://geneontology.org/) and KEGG (www.kegg.jp/kegg) enrichment analysis of the predicted target genes was performed using the clusterProfiler R package^63^.

### Validations of the selected DE miRNAs and their targets by qRT-PCR

To validate some of the selected DE miRNAs and their targets, their relative expressions were determined by performing qPCR analysis. From the collected samples, miRNA and total RNA was isolated using the Easy pure miRNA kit (Transgen, Beijing, China) and the TransZol Up reagent (Transgen), respectively, according to the manufacturer’s instructions. First strand cDNA from the miRNA and the total RNA was amplified by using the miRcute Plus miR-first strand cDNA kit (Tiangen, Shenzhen, China) and the Transcript one-step gDNA removal and cDNA synthesis supermix kit (Transgen), respectively. The miRNA-specific forward primers and a universal reverse primer, and gene-specific primer pairs were used to determine the miRNA and target expression analysis, along with the SYBR Green PCR mix (Transgen) on the ABI 7500 real-time PCR system (Applied Biosystems, CA, USA). Three independent biological samples for each reaction, and three technical replicates for each biological sample, were used for the qPCR analysis. The *U6* gene was used as the internal reference gene for evaluating the miRNA relative expression levels, whereas the constitutively expressed housekeeping gene *OsUbq* from was used as an endogenous control for the targets^64^. The relative expression was evaluated using the 2^−ΔΔCt^ method^65^.

### Statistical analysis

The statistical analyses of the relative expressions were carried out using Data Processing System software^66^. Data are reported as mean ± SE. Expressions of miRNAs and the targets were analyzed by student’s *t*-test. The statistical significance level was set for *P* values < 0.05 or 0.01.

## Supplementary information

Supplementary Legends.

Supplementary Figure S1.

Supplementary Figure S2.

Supplementary Tables.
