# Supplementary material for: Identification and analysis of miRNAs in IR56 rice in response to BPH infestations of different virulence levels
Source: Sci Rep. 2020 Nov 5;10:19093. doi: 10.1038/s41598-020-76198-9 (PMC7645692; doi:10.1038/s41598-020-76198-9)
Supplement: Supplementary file 1 — Supplementary Legends. [file 41598_2020_76198_MOESM1_ESM.docx]

**Figure S1. Annotations of small RNA sequencing results in the IR56 rice.**

**Figure S2. The Pearson correlation analysis, among different IR56 rice sRNA libraries.**
