# Supplementary figures and images for: Identification and analysis of miRNAs in IR56 rice in response to BPH infestations of different virulence levels
Source: Sci Rep. 2020 Nov 5;10:19093. doi: 10.1038/s41598-020-76198-9 (PMC7645692; doi:10.1038/s41598-020-76198-9)

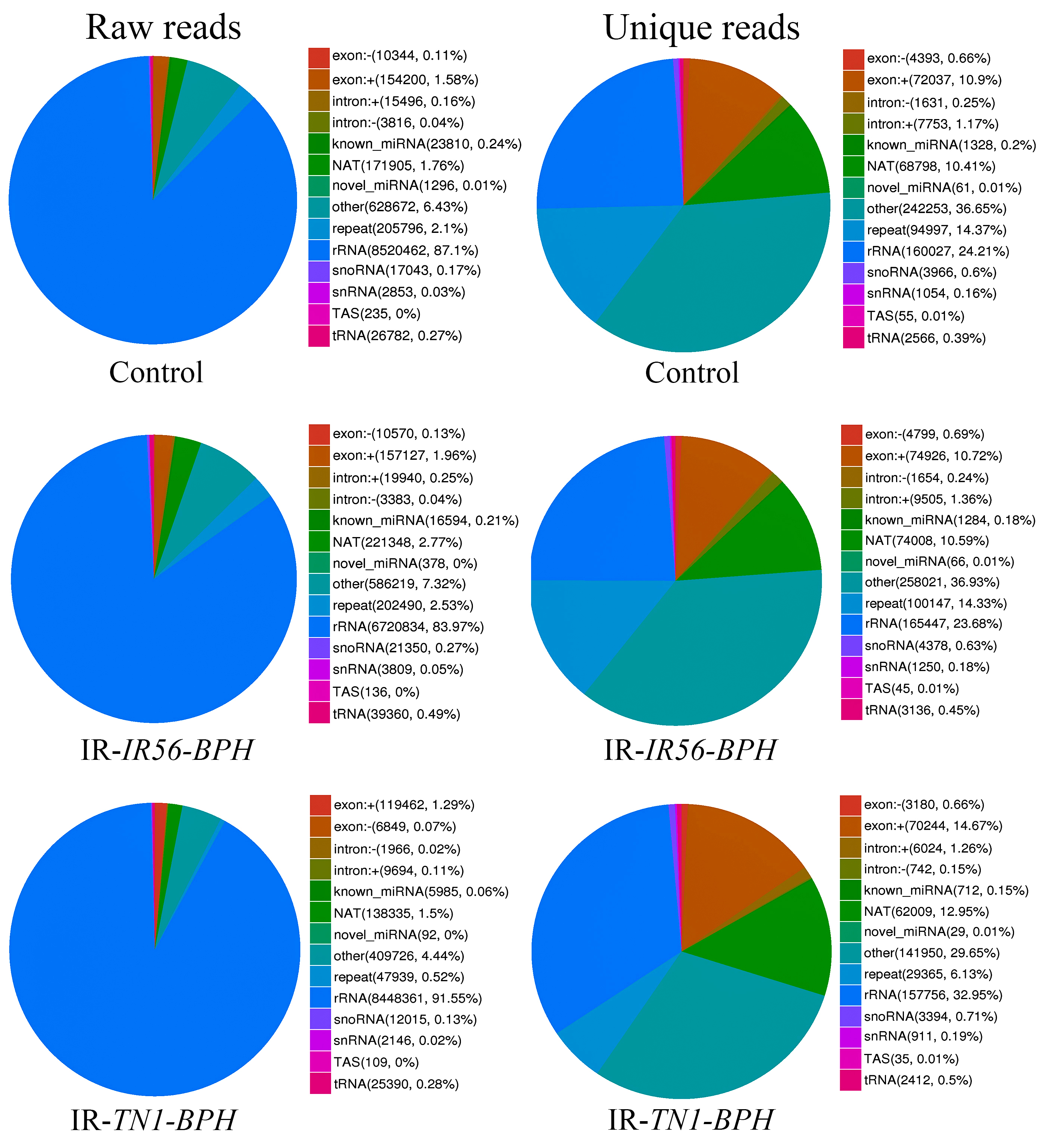

Supplement: Supplementary file 2 — Supplementary Figure S1. [file 41598_2020_76198_MOESM2_ESM.jpg]

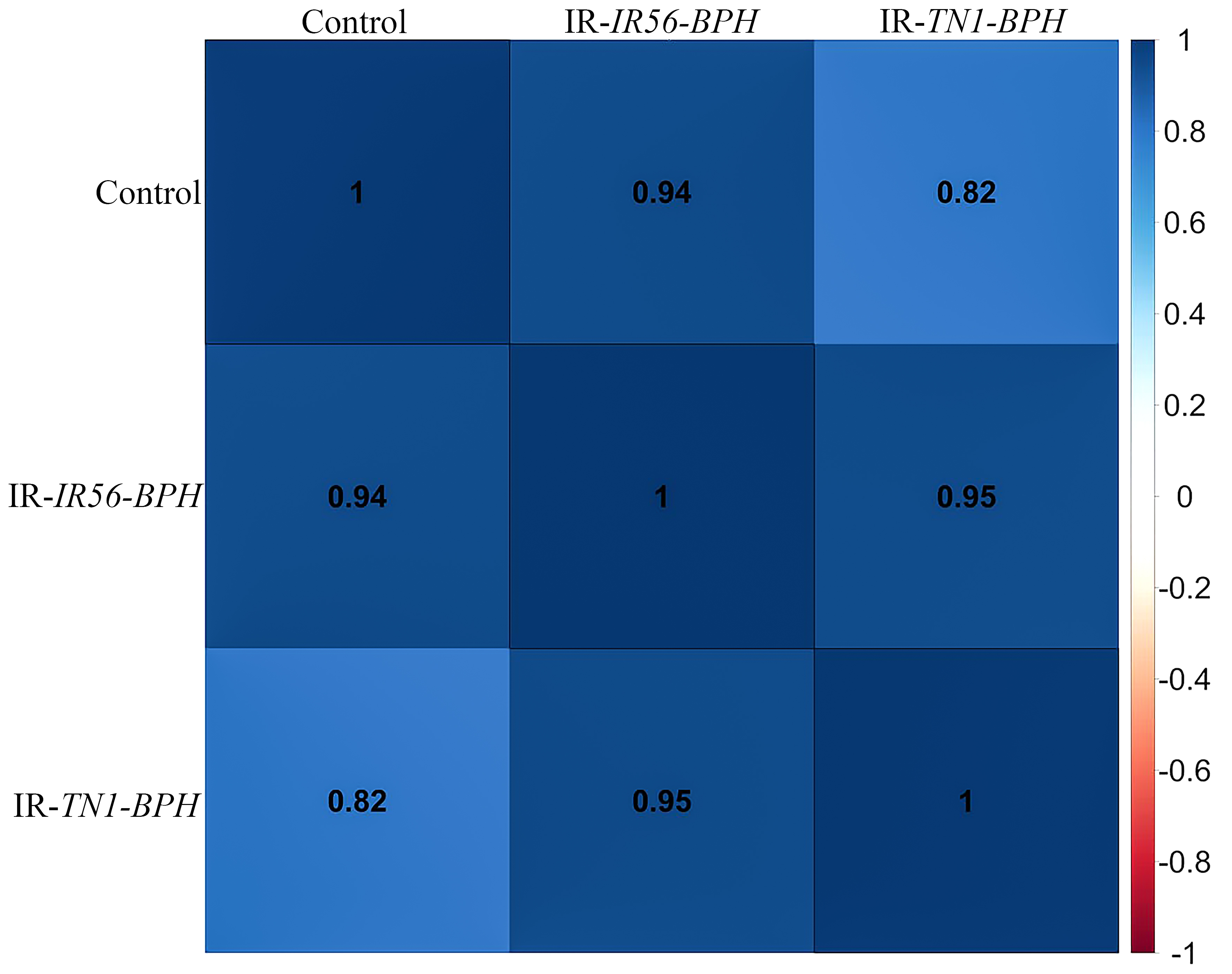

Supplement: Supplementary file 3 — Supplementary Figure S2. [file 41598_2020_76198_MOESM3_ESM.jpg]
