# Supplementary material for: Identification and analysis of miRNAs in IR56 rice in response to BPH infestations of different virulence levels
Source: Sci Rep. 2020 Nov 5;10:19093. doi: 10.1038/s41598-020-76198-9 (PMC7645692; doi:10.1038/s41598-020-76198-9)
Supplement: Supplementary file 4 — Supplementary Tables. [file 41598_2020_76198_MOESM4_ESM.docx]

**Identification and analysis of miRNAs in IR56 rice in response to BPH infestations of different virulence levels**

Satyabrata Nanda, San-Yue Yuan, Feng-Xia Lai, Wei-Xia Wang, Qiang Fu*, Pin-Jun Wan*

**Table S1. List of the miRNA families identified in rice and the belonging miRNAs.­­**

| **miRNA family** | **miRNAs identified in the study** | **miRNA family** | **miRNAs identified in the study** |
| --- | --- | --- | --- |
| **MIR1440** | osa-MIR1440a  osa-MIR1440b | **MIR5534** | osa-MIR5534a |
| **MIR2873** | osa-MIR2873a  osa-MIR2873b  osa-MIR2873c | **MIR171** | osa-MIR171h |
| **MIR812** | osa-MIR812a osa-MIR812f osa-MIR812g osa-MIR812k osa-MIR812n osa-MIR812o osa-MIR812p osa-MIR812q osa-MIR812r osa-MIR812s osa-MIR812v | **MIR814** | osa-MIR814a |
| **MIR168** | osa-MIR168a | **MIR171** | osa-MIR171a osa-MIR171b osa-MIR171c osa-MIR171d osa-MIR171e osa-MIR171f osa-MIR171i |
| **MIR2871** | osa-MIR2871a osa-MIR2871b | **MIR530** | osa-MIR530 |
| **MIR439** | osa-MIR439a | **MIR820** | osa-MIR820a |
| **MIR169** | osa-MIR169a osa-MIR169i osa-MIR169r | **MIR529** | osa-MIR529a osa-MIR529b |
| **MIR5079** | osa-MIR5079a | **MIR1846** | osa-MIR1846a |
| **MIR159** | osa-MIR159a osa-MIR159c osa-MIR159f | **MIR166** | osa-MIR166a osa-MIR166b osa-MIR166c osa-MIR166d osa-MIR166e osa-MIR166g osa-MIR166h osa-MIR166i osa-MIR166j osa-MIR166k osa-MIR166l osa-MIR166m |
| **MIR319** | osa-MIR319a | **MIR1862** | osa-MIR1862d osa-MIR1862e |
| **MIR821** | osa-MIR821a | **MIR1319** | osa-MIR1319a |
| **MIR160** | osa-MIR160a osa-MIR160c osa-MIR160d osa-MIR160e osa-MIR160f | **MIR444** | osa-MIR444a osa-MIR444b osa-MIR444d |
| **MIR160** | osa- MIR160a | **MIR535** | osa-MIR535 |
| **MIR393** | osa-MIR393b | **MIR3980** | osa-MIR3980a |
| **MIR531** | osa-MIR531a osa-MIR531b | **MIR399** | osa-MIR399a osa-MIR399d osa-MIR399i osa-MIR399j |
| **MIR5539** | osa-MIR5539a | **MIR1437** | osa-MIR1437a osa-MIR1437b |
| **MIR437** | osa-MIR437 | **MIR1878** | osa-MIR1878 |
| **MIR810** | osa-MIR810a osa-MIR810b | **MIR827** | osa-MIR827 |
| **MIR2863** | osa-MIR2863a osa-MIR2863b osa-MIR2863c | **MIR1436** | osa-MIR1436 |
| **MIR815** | osa-MIR815a | **MIR818** | osa-MIR818a |
| **MIR1863** | osa-MIR1863b osa-MIR1863c | **MIR395** | osa-MIR395b osa-MIR395c osa-MIR395o osa-MIR395w |
| **MIR390** | osa-MIR390 | **MIR156** | osa-MIR156a osa-MIR156b osa-MIR156c osa-MIR156f osa-MIR156j osa-MIR156k osa-MIR156l |
| **MIR397** | osa-miR397a, osa-miR397b | **MIR172** | osa-miR172a, osa-miR172d-5p |
| **MIR5160** | osa-MIR5160 | **MIR162** | osa-MIR162a osa-MIR162b |
|  | osa-MIR5540 | **MIR1428** | osa-MIR1428e |
| **MIR5148** | osa-MIR5148a | **MIR398** | osa-MIR398b |
| **MIR396** | osa-MIR396a osa-MIR396c osa-MIR396e osa-MIR396f osa-MIR396g | **MIR528** | osa-MIR528 |
| **MIR167** | osa-MIR167a osa-MIR167c osa-MIR167d osa-MIR167e osa-MIR167h | **MIR1859** | osa-MIR1859 |
| **MIR408** | osa-MIR408 | **MIR1883** | osa-MIR1883a |
| **MIR818** | osa-MIR818f | **MIR5824** | osa-MIR5824 |
| **MIR435** | osa-miR435 |  |  |

**Table S2. List of the exclusive miRNA detected in specific libraries.­­**

| **IR-TN1-BPH** | **IR-IR56-BPH** | **Control (CK, no BPH)** |
| --- | --- | --- |
| osa-miR1432-3p | osa-miR11340-3p | osa-miR11338-5p |
| osa-miR156k | osa-miR156c-3p | osa-miR1319a |
| osa-miR166g-5p | osa-miR166e-3p | osa-miR1428e-3p |
| osa-miR1882e-3p | osa-miR1852 | osa-miR1429-5p |
| osa-miR2118g | osa-miR1857-5p | osa-miR159a.2 |
| osa-miR435 | osa-miR1870-3p | osa-miR164c |
|  | osa-miR2055 | osa-miR169r-5p |
|  | osa-miR2864.1 | osa-miR1857-3p |
|  | osa-miR2873a | osa-miR1861g |
|  | osa-miR3981-3p | osa-miR1862f |
|  | osa-miR5144-3p | osa-miR1874-3p |
|  | osa-miR5159 | osa-miR1874-5p |
|  | osa-miR530-5p | osa-miR1875 |
|  | osa-miR5337a | osa-miR2872 |
|  | osa-miR535-3p | osa-miR2879 |
|  | osa-miR5538 | osa-miR3980a-3p |
|  | osa-miR5801b | osa-miR437 |
|  | osa-miR6246 | osa-miR439a |
|  | osa-miR7692-3p | osa-miR444a-3p.2 |
|  | osa-miR812s | osa-miR5073 |
|  | novel_128 | osa-miR5151 |
|  | novel_16 | osa-miR5337b |
|  | novel_47 | osa-miR5493 |
|  | novel_52 | osa-miR5801c-3p |
|  |  | osa-miR5814 |
|  |  | osa-miR6251 |
|  |  | osa-miR812a |
|  |  | osa-miR812n-3p |
|  |  | osa-miR812v |
|  |  | osa-miR817 |
|  |  | novel_139 |

**Table S3. List of the DE miRNA identified in IR56 rice.**

| **sRNA** | **IR56_IR** | **IR56_CK** | **log2.Fold_change.** | **p.value** | **q.value** |
| --- | --- | --- | --- | --- | --- |
| **IR56-IR vs IR56-CK** | | | | | |
| novel_117 | 2766.593352 | 9445.761826 | -1.7716 | 0 | 0 |
| novel_121 | 2662.193603 | 38352.64098 | -3.8486 | 0 | 0 |
| osa-miR1861a | 16390.76061 | 35409.74031 | -1.1113 | 0 | 0 |
| osa-miR1861b | 213393.0872 | 461940.4728 | -1.1142 | 0 | 0 |
| osa-miR1861h | 2505.593979 | 20980.0338 | -3.0658 | 0 | 0 |
| osa-miR399d | 2087.994983 | 7642.048513 | -1.8718 | 0 | 0 |
| osa-miR397b | 1774.795735 | 427.1952585 | 2.0547 | 1.05E-290 | 4.15E-290 |
| osa-miR531a | 2714.393478 | 1281.585775 | 1.0827 | 1.17E-233 | 3.88E-233 |
| osa-miR531b | 2714.393478 | 1281.585775 | 1.0827 | 1.17E-233 | 3.88E-233 |
| osa-miR395b | 104.3997491 | 1423.984195 | -3.7697 | 5.86E-227 | 1.85E-226 |
| osa-miR1861c | 782.9981185 | 3227.697508 | -2.0434 | 3.73E-216 | 1.12E-215 |
| osa-miR408-3p | 2140.194857 | 949.3227966 | 1.1728 | 2.08E-200 | 5.96E-200 |
| osa-miR5150-5p | 835.1979931 | 94.93227966 | 3.1371 | 1.72E-193 | 4.52E-193 |
| osa-miR528-5p | 1513.796362 | 522.1275381 | 1.5357 | 2.93E-187 | 7.38E-187 |
| osa-miR530-5p | 1200.597115 | 0 | 11.23 | 1.07E-178 | 2.60E-178 |
| osa-miR5153 | 1826.99561 | 806.9243771 | 1.179 | 2.81E-172 | 6.55E-172 |
| osa-miR11340-5p | 939.5977422 | 189.8645593 | 2.3071 | 1.13E-171 | 2.55E-171 |
| novel_14 | 626.3984948 | 47.46613983 | 3.7221 | 1.23E-160 | 2.67E-160 |
| osa-miR1861d | 313.1992474 | 1613.848754 | -2.3654 | 3.09E-140 | 6.29E-140 |
| osa-miR11339-5p | 1670.395986 | 806.9243771 | 1.0497 | 5.63E-140 | 1.11E-139 |
| osa-miR167d-3p | 1043.997491 | 332.2629788 | 1.6517 | 1.80E-139 | 3.44E-139 |
| osa-miR398b | 1565.996237 | 759.4582373 | 1.044 | 1.27E-130 | 2.36E-130 |
| osa-miR11342-3p | 1043.997491 | 3037.832949 | -1.5409 | 1.41E-114 | 2.46E-114 |
| osa-miR1860-3p | 469.7988711 | 47.46613983 | 3.3071 | 2.13E-113 | 3.62E-113 |
| osa-miR1320-5p | 626.3984948 | 142.3984195 | 2.1371 | 1.27E-107 | 2.05E-107 |
| osa-miR397a | 835.1979931 | 284.796839 | 1.5522 | 2.75E-105 | 4.34E-105 |
| osa-miR393b-3p | 417.5989966 | 47.46613983 | 3.1371 | 9.91E-98 | 1.49E-97 |
| osa-miR169i-3p | 991.7976168 | 427.1952585 | 1.2152 | 2.50E-97 | 3.66E-97 |
| osa-miR3980a-5p | 0 | 664.5259576 | -10.376 | 5.37E-97 | 7.69E-97 |
| osa-miR2863c | 365.399122 | 47.46613983 | 2.9445 | 3.61E-82 | 4.84E-82 |
| novel_75 | 521.9987457 | 142.3984195 | 1.8741 | 8.90E-80 | 1.12E-79 |
| osa-miR2878-5p | 521.9987457 | 142.3984195 | 1.8741 | 8.90E-80 | 1.12E-79 |
| osa-miR11337-3p | 1148.397241 | 2895.43453 | -1.3342 | 9.64E-78 | 1.19E-77 |
| novel_43 | 52.19987457 | 522.1275381 | -3.3223 | 4.44E-74 | 5.38E-74 |
| osa-miR166a-5p | 417.5989966 | 94.93227966 | 2.1371 | 2.24E-72 | 2.66E-72 |
| novel_45 | 730.798244 | 332.2629788 | 1.1371 | 1.63E-67 | 1.87E-67 |
| osa-miR167h-3p | 469.7988711 | 142.3984195 | 1.7221 | 2.02E-66 | 2.24E-66 |
| osa-miR6246 | 313.1992474 | 0 | 9.2909 | 9.96E-65 | 1.05E-64 |
| osa-miR812s | 313.1992474 | 0 | 9.2909 | 9.96E-65 | 1.05E-64 |
| osa-miR171d-5p | 365.399122 | 94.93227966 | 1.9445 | 2.35E-58 | 2.39E-58 |
| osa-miR171b | 626.3984948 | 284.796839 | 1.1371 | 3.98E-58 | 3.98E-58 |
| novel_52 | 260.9993728 | 0 | 9.0279 | 3.46E-56 | 3.35E-56 |
| osa-miR159f | 260.9993728 | 0 | 9.0279 | 3.46E-56 | 3.35E-56 |
| osa-miR11338-3p | 1513.796362 | 3275.163648 | -1.1134 | 4.27E-54 | 4.02E-54 |
| osa-miR11341-5p | 260.9993728 | 47.46613983 | 2.4591 | 1.08E-51 | 9.74E-52 |
| osa-miR169i-5p.2 | 260.9993728 | 47.46613983 | 2.4591 | 1.08E-51 | 9.74E-52 |
| osa-miR7694-3p | 260.9993728 | 47.46613983 | 2.4591 | 1.08E-51 | 9.74E-52 |
| osa-miR1861e | 156.5996237 | 664.5259576 | -2.0852 | 1.26E-47 | 1.04E-47 |
| novel_128 | 208.7994983 | 0 | 8.706 | 3.40E-47 | 2.68E-47 |
| novel_47 | 208.7994983 | 0 | 8.706 | 3.40E-47 | 2.68E-47 |
| osa-miR11340-3p | 208.7994983 | 0 | 8.706 | 3.40E-47 | 2.68E-47 |
| osa-miR535-3p | 208.7994983 | 0 | 8.706 | 3.40E-47 | 2.68E-47 |
| osa-miR5505 | 104.3997491 | 522.1275381 | -2.3223 | 2.90E-45 | 2.26E-45 |
| novel_72 | 313.1992474 | 94.93227966 | 1.7221 | 7.08E-45 | 5.44E-45 |
| osa-miR5493 | 0 | 237.3306991 | -8.8908 | 5.91E-43 | 4.49E-43 |
| osa-miR5082 | 365.399122 | 142.3984195 | 1.3595 | 2.46E-41 | 1.84E-41 |
| osa-miR166b-5p | 156.5996237 | 0 | 8.2909 | 1.28E-37 | 9.20E-38 |
| osa-miR1865-3p | 156.5996237 | 0 | 8.2909 | 1.28E-37 | 9.20E-38 |
| osa-miR5337a | 156.5996237 | 0 | 8.2909 | 1.28E-37 | 9.20E-38 |
| novel_44 | 208.7994983 | 47.46613983 | 2.1371 | 4.47E-37 | 3.13E-37 |
| osa-miR5802 | 208.7994983 | 47.46613983 | 2.1371 | 4.47E-37 | 3.13E-37 |
| novel_138 | 52.19987457 | 332.2629788 | -2.6702 | 3.00E-36 | 2.05E-36 |
| osa-miR399a | 52.19987457 | 332.2629788 | -2.6702 | 3.00E-36 | 2.05E-36 |
| osa-miR1875 | 0 | 189.8645593 | -8.5688 | 7.63E-36 | 5.12E-36 |
| osa-miR399i | 0 | 189.8645593 | -8.5688 | 7.63E-36 | 5.12E-36 |
| osa-miR169r-5p | 0 | 142.3984195 | -8.1538 | 2.55E-28 | 1.62E-28 |
| osa-miR444a-3p.2 | 0 | 142.3984195 | -8.1538 | 2.55E-28 | 1.62E-28 |
| osa-miR817 | 0 | 142.3984195 | -8.1538 | 2.55E-28 | 1.62E-28 |
| osa-miR396c-3p | 52.19987457 | 284.796839 | -2.4478 | 1.34E-27 | 8.46E-28 |
| novel_16 | 104.3997491 | 0 | 7.706 | 3.16E-27 | 1.90E-27 |
| osa-miR156c-3p | 104.3997491 | 0 | 7.706 | 3.16E-27 | 1.90E-27 |
| osa-miR166e-3p | 104.3997491 | 0 | 7.706 | 3.16E-27 | 1.90E-27 |
| osa-miR1870-3p | 104.3997491 | 0 | 7.706 | 3.16E-27 | 1.90E-27 |
| osa-miR5538 | 104.3997491 | 0 | 7.706 | 3.16E-27 | 1.90E-27 |
| osa-miR171c-5p | 156.5996237 | 47.46613983 | 1.7221 | 2.83E-23 | 1.67E-23 |
| novel_134 | 156.5996237 | 474.6613983 | -1.5998 | 6.94E-21 | 3.91E-21 |
| osa-miR5083 | 156.5996237 | 474.6613983 | -1.5998 | 6.94E-21 | 3.91E-21 |
| osa-miR1846a-5p | 208.7994983 | 94.93227966 | 1.1371 | 1.73E-20 | 9.54E-21 |
| osa-miR444d.3 | 208.7994983 | 94.93227966 | 1.1371 | 1.73E-20 | 9.54E-21 |
| novel_139 | 0 | 94.93227966 | -7.5688 | 3.10E-20 | 1.56E-20 |
| osa-miR1319a | 0 | 94.93227966 | -7.5688 | 3.10E-20 | 1.56E-20 |
| osa-miR1428e-3p | 0 | 94.93227966 | -7.5688 | 3.10E-20 | 1.56E-20 |
| osa-miR1429-5p | 0 | 94.93227966 | -7.5688 | 3.10E-20 | 1.56E-20 |
| osa-miR156l-5p | 0 | 94.93227966 | -7.5688 | 3.10E-20 | 1.56E-20 |
| osa-miR159a.2 | 0 | 94.93227966 | -7.5688 | 3.10E-20 | 1.56E-20 |
| osa-miR164e | 0 | 94.93227966 | -7.5688 | 3.10E-20 | 1.56E-20 |
| osa-miR1861g | 0 | 94.93227966 | -7.5688 | 3.10E-20 | 1.56E-20 |
| osa-miR1874-3p | 0 | 94.93227966 | -7.5688 | 3.10E-20 | 1.56E-20 |
| osa-miR5151 | 0 | 94.93227966 | -7.5688 | 3.10E-20 | 1.56E-20 |
| osa-miR5539a | 0 | 94.93227966 | -7.5688 | 3.10E-20 | 1.56E-20 |
| osa-miR5813 | 52.19987457 | 237.3306991 | -2.1848 | 1.68E-19 | 8.26E-20 |
| osa-miR11343-5p | 104.3997491 | 332.2629788 | -1.6702 | 2.01E-16 | 9.66E-17 |
| osa-miR1852 | 52.19987457 | 0 | 6.706 | 1.54E-15 | 6.78E-16 |
| osa-miR1857-5p | 52.19987457 | 0 | 6.706 | 1.54E-15 | 6.78E-16 |
| osa-miR2055 | 52.19987457 | 0 | 6.706 | 1.54E-15 | 6.78E-16 |
| osa-miR2864.1 | 52.19987457 | 0 | 6.706 | 1.54E-15 | 6.78E-16 |
| osa-miR2867-3p | 52.19987457 | 0 | 6.706 | 1.54E-15 | 6.78E-16 |
| osa-miR2873a | 52.19987457 | 0 | 6.706 | 1.54E-15 | 6.78E-16 |
| osa-miR3981-3p | 52.19987457 | 0 | 6.706 | 1.54E-15 | 6.78E-16 |
| osa-miR5144-3p | 52.19987457 | 0 | 6.706 | 1.54E-15 | 6.78E-16 |
| osa-miR5159 | 52.19987457 | 0 | 6.706 | 1.54E-15 | 6.78E-16 |
| osa-miR5508 | 52.19987457 | 0 | 6.706 | 1.54E-15 | 6.78E-16 |
| osa-miR5801b | 52.19987457 | 0 | 6.706 | 1.54E-15 | 6.78E-16 |
| osa-miR7692-3p | 52.19987457 | 0 | 6.706 | 1.54E-15 | 6.78E-16 |
| osa-miR5340 | 208.7994983 | 522.1275381 | -1.3223 | 4.89E-15 | 2.12E-15 |
| osa-miR5540 | 52.19987457 | 189.8645593 | -1.8629 | 3.44E-12 | 1.45E-12 |
| osa-miR6250 | 52.19987457 | 189.8645593 | -1.8629 | 3.44E-12 | 1.45E-12 |
| osa-miR11338-5p | 0 | 47.46613983 | -6.5688 | 2.72E-11 | 1.02E-11 |
| osa-miR164c | 0 | 47.46613983 | -6.5688 | 2.72E-11 | 1.02E-11 |
| osa-miR1857-3p | 0 | 47.46613983 | -6.5688 | 2.72E-11 | 1.02E-11 |
| osa-miR1862f | 0 | 47.46613983 | -6.5688 | 2.72E-11 | 1.02E-11 |
| osa-miR1874-5p | 0 | 47.46613983 | -6.5688 | 2.72E-11 | 1.02E-11 |
| osa-miR2872 | 0 | 47.46613983 | -6.5688 | 2.72E-11 | 1.02E-11 |
| osa-miR2879 | 0 | 47.46613983 | -6.5688 | 2.72E-11 | 1.02E-11 |
| osa-miR3980a-3p | 0 | 47.46613983 | -6.5688 | 2.72E-11 | 1.02E-11 |
| osa-miR437 | 0 | 47.46613983 | -6.5688 | 2.72E-11 | 1.02E-11 |
| osa-miR439a | 0 | 47.46613983 | -6.5688 | 2.72E-11 | 1.02E-11 |
| osa-miR5072 | 0 | 47.46613983 | -6.5688 | 2.72E-11 | 1.02E-11 |
| osa-miR5073 | 0 | 47.46613983 | -6.5688 | 2.72E-11 | 1.02E-11 |
| osa-miR5337b | 0 | 47.46613983 | -6.5688 | 2.72E-11 | 1.02E-11 |
| osa-miR5801c-3p | 0 | 47.46613983 | -6.5688 | 2.72E-11 | 1.02E-11 |
| osa-miR5814 | 0 | 47.46613983 | -6.5688 | 2.72E-11 | 1.02E-11 |
| osa-miR6251 | 0 | 47.46613983 | -6.5688 | 2.72E-11 | 1.02E-11 |
| osa-miR812a | 0 | 47.46613983 | -6.5688 | 2.72E-11 | 1.02E-11 |
| osa-miR812n-3p | 0 | 47.46613983 | -6.5688 | 2.72E-11 | 1.02E-11 |
| osa-miR812v | 0 | 47.46613983 | -6.5688 | 2.72E-11 | 1.02E-11 |
| osa-miR1429-3p | 104.3997491 | 47.46613983 | 1.1371 | 5.37E-11 | 1.94E-11 |
| osa-miR1863b | 104.3997491 | 47.46613983 | 1.1371 | 5.37E-11 | 1.94E-11 |
| osa-miR5788 | 104.3997491 | 47.46613983 | 1.1371 | 5.37E-11 | 1.94E-11 |
| osa-miR7693-5p | 104.3997491 | 47.46613983 | 1.1371 | 5.37E-11 | 1.94E-11 |
| osa-miR810b.2 | 104.3997491 | 47.46613983 | 1.1371 | 5.37E-11 | 1.94E-11 |
| osa-miR5076 | 104.3997491 | 284.796839 | -1.4478 | 9.75E-11 | 3.51E-11 |
| osa-miR11336-5p | 208.7994983 | 474.6613983 | -1.1848 | 1.18E-10 | 4.22E-11 |
| novel_107 | 208.7994983 | 427.1952585 | -1.0328 | 6.38E-07 | 2.19E-07 |
| novel_66 | 52.19987457 | 142.3984195 | -1.4478 | 4.76E-06 | 1.59E-06 |
| osa-miR1425-3p | 52.19987457 | 142.3984195 | -1.4478 | 4.76E-06 | 1.59E-06 |
| osa-miR171a | 52.19987457 | 142.3984195 | -1.4478 | 4.76E-06 | 1.59E-06 |
| osa-miR1868 | 52.19987457 | 142.3984195 | -1.4478 | 4.76E-06 | 1.59E-06 |
| **IR56-TN1 vs IR56-CK** | | | | | |
| novel_117 | 2247.947847 | 10037.52434 | -2.1587 | 0 | 0 |
| novel_121 | 1586.786715 | 40755.37522 | -4.6828 | 0 | 0 |
| osa-miR156a | 12033.13259 | 5245.741365 | 1.1978 | 0 | 0 |
| osa-miR166d-5p | 1719.018941 | 403.5185665 | 2.0909 | 0 | 0 |
| osa-miR1861a | 14148.84821 | 37628.10633 | -1.4111 | 0 | 0 |
| osa-miR1861b | 140033.9276 | 490880.3362 | -1.8096 | 0 | 0 |
| osa-miR1861h | 3173.57343 | 22294.4008 | -2.8125 | 0 | 0 |
| osa-miR1862e | 5685.98573 | 17401.73818 | -1.6137 | 0 | 0 |
| osa-miR393b-3p | 1057.85781 | 50.43982082 | 4.3904 | 0 | 0 |
| osa-miR396g | 11107.50701 | 4741.343157 | 1.2282 | 0 | 0 |
| osa-miR408-3p | 2512.412299 | 1008.796416 | 1.3164 | 0 | 0 |
| osa-miR528-5p | 3305.805657 | 554.838029 | 2.5749 | 0 | 0 |
| osa-miR827 | 5157.056824 | 2320.231758 | 1.1523 | 0 | 0 |
| novel_14 | 925.6255839 | 50.43982082 | 4.1978 | 1.27E-270 | 6.16E-271 |
| osa-miR1423-5p | 132.2322263 | 1714.953908 | -3.697 | 1.43E-242 | 6.32E-243 |
| osa-miR1861c | 793.3933576 | 3429.907816 | -2.1121 | 1.12E-197 | 4.09E-198 |
| novel_132 | 396.6966788 | 2219.352116 | -2.484 | 8.94E-176 | 2.76E-176 |
| novel_99 | 793.3933576 | 3177.708712 | -2.0019 | 1.73E-163 | 5.03E-164 |
| osa-miR164e | 661.1611313 | 100.8796416 | 2.7124 | 9.30E-156 | 2.63E-156 |
| osa-miR5083 | 1190.090036 | 504.3982082 | 1.2384 | 8.32E-146 | 2.23E-146 |
| osa-miR1860-3p | 528.9289051 | 50.43982082 | 3.3904 | 8.84E-143 | 2.31E-143 |
| osa-miR399j | 528.9289051 | 100.8796416 | 2.3904 | 2.92E-114 | 7.24E-115 |
| osa-miR5801c-5p | 396.6966788 | 1815.833549 | -2.1945 | 4.65E-114 | 1.13E-114 |
| osa-miR169i-5p.2 | 396.6966788 | 50.43982082 | 2.9754 | 5.31E-100 | 1.26E-100 |
| osa-miR1861e | 0 | 706.1574914 | -10.464 | 2.75E-99 | 6.37E-100 |
| osa-miR167h-3p | 528.9289051 | 151.3194625 | 1.8055 | 2.68E-91 | 5.93E-92 |
| osa-miR5505 | 0 | 554.838029 | -10.116 | 3.14E-82 | 6.39E-83 |
| osa-miR159f | 396.6966788 | 0 | 9.6319 | 3.41E-81 | 6.55E-82 |
| osa-miR2118g | 396.6966788 | 0 | 9.6319 | 3.41E-81 | 6.55E-82 |
| osa-miR435 | 396.6966788 | 0 | 9.6319 | 3.41E-81 | 6.55E-82 |
| novel_134 | 0 | 504.3982082 | -9.9784 | 2.75E-76 | 5.00E-77 |
| osa-miR2871a-3p | 0 | 504.3982082 | -9.9784 | 2.75E-76 | 5.00E-77 |
| osa-miR5150-5p | 396.6966788 | 100.8796416 | 1.9754 | 2.93E-74 | 5.24E-75 |
| osa-miR399i | 528.9289051 | 201.7592833 | 1.3904 | 7.17E-73 | 1.24E-73 |
| novel_107 | 0 | 453.9583874 | -9.8264 | 3.42E-70 | 5.71E-71 |
| osa-miR166b-5p | 264.4644525 | 0 | 9.0469 | 1.87E-59 | 2.97E-60 |
| osa-miR11341-5p | 264.4644525 | 50.43982082 | 2.3904 | 4.58E-58 | 6.96E-59 |
| osa-miR1860-5p | 264.4644525 | 50.43982082 | 2.3904 | 4.58E-58 | 6.96E-59 |
| osa-miR5788 | 264.4644525 | 50.43982082 | 2.3904 | 4.58E-58 | 6.96E-59 |
| novel_138 | 0 | 353.0787457 | -9.4638 | 1.83E-57 | 2.70E-58 |
| osa-miR11343-5p | 0 | 353.0787457 | -9.4638 | 1.83E-57 | 2.70E-58 |
| novel_75 | 396.6966788 | 151.3194625 | 1.3904 | 4.16E-55 | 5.97E-56 |
| osa-miR1320-5p | 396.6966788 | 151.3194625 | 1.3904 | 4.16E-55 | 5.97E-56 |
| novel_126 | 0 | 302.6389249 | -9.2415 | 8.96E-51 | 1.23E-51 |
| osa-miR167e-3p | 0 | 302.6389249 | -9.2415 | 8.96E-51 | 1.23E-51 |
| osa-miR166k-5p | 0 | 252.1991041 | -8.9784 | 8.15E-44 | 9.99E-45 |
| osa-miR1850.3 | 0 | 252.1991041 | -8.9784 | 8.15E-44 | 9.99E-45 |
| osa-miR1870-5p | 0 | 252.1991041 | -8.9784 | 8.15E-44 | 9.99E-45 |
| osa-miR5493 | 0 | 252.1991041 | -8.9784 | 8.15E-44 | 9.99E-45 |
| osa-miR5813 | 0 | 252.1991041 | -8.9784 | 8.15E-44 | 9.99E-45 |
| osa-miR395b | 528.9289051 | 1513.194625 | -1.5165 | 4.91E-40 | 5.88E-41 |
| novel_72 | 264.4644525 | 100.8796416 | 1.3904 | 2.54E-37 | 2.97E-38 |
| osa-miR1875 | 0 | 201.7592833 | -8.6565 | 1.58E-36 | 1.79E-37 |
| osa-miR5513 | 0 | 201.7592833 | -8.6565 | 1.58E-36 | 1.79E-37 |
| osa-miR6250 | 0 | 201.7592833 | -8.6565 | 1.58E-36 | 1.79E-37 |
| osa-miR1432-3p | 132.2322263 | 0 | 8.0469 | 1.49E-34 | 1.56E-35 |
| osa-miR156k | 132.2322263 | 0 | 8.0469 | 1.49E-34 | 1.56E-35 |
| osa-miR166g-5p | 132.2322263 | 0 | 8.0469 | 1.49E-34 | 1.56E-35 |
| osa-miR1865-3p | 132.2322263 | 0 | 8.0469 | 1.49E-34 | 1.56E-35 |
| osa-miR1882e-3p | 132.2322263 | 0 | 8.0469 | 1.49E-34 | 1.56E-35 |
| osa-miR2867-3p | 132.2322263 | 0 | 8.0469 | 1.49E-34 | 1.56E-35 |
| osa-miR5508 | 132.2322263 | 0 | 8.0469 | 1.49E-34 | 1.56E-35 |
| osa-miR1861d | 661.1611313 | 1714.953908 | -1.3751 | 4.99E-34 | 5.18E-35 |
| novel_43 | 132.2322263 | 554.838029 | -2.069 | 4.16E-32 | 4.19E-33 |
| osa-miR5340 | 132.2322263 | 554.838029 | -2.069 | 4.16E-32 | 4.19E-33 |
| osa-miR171i-3p | 264.4644525 | 857.4769539 | -1.697 | 3.47E-31 | 3.40E-32 |
| osa-miR2106 | 264.4644525 | 857.4769539 | -1.697 | 3.47E-31 | 3.40E-32 |
| osa-miR5153 | 264.4644525 | 857.4769539 | -1.697 | 3.47E-31 | 3.40E-32 |
| novel_66 | 0 | 151.3194625 | -8.2415 | 8.02E-29 | 6.92E-30 |
| osa-miR169r-5p | 0 | 151.3194625 | -8.2415 | 8.02E-29 | 6.92E-30 |
| osa-miR171a | 0 | 151.3194625 | -8.2415 | 8.02E-29 | 6.92E-30 |
| osa-miR172a | 0 | 151.3194625 | -8.2415 | 8.02E-29 | 6.92E-30 |
| osa-miR1850.1 | 0 | 151.3194625 | -8.2415 | 8.02E-29 | 6.92E-30 |
| osa-miR1863b.2 | 0 | 151.3194625 | -8.2415 | 8.02E-29 | 6.92E-30 |
| osa-miR1868 | 0 | 151.3194625 | -8.2415 | 8.02E-29 | 6.92E-30 |
| osa-miR2880 | 0 | 151.3194625 | -8.2415 | 8.02E-29 | 6.92E-30 |
| osa-miR444a-3p.2 | 0 | 151.3194625 | -8.2415 | 8.02E-29 | 6.92E-30 |
| osa-miR528-3p | 0 | 151.3194625 | -8.2415 | 8.02E-29 | 6.92E-30 |
| osa-miR812n-5p | 0 | 151.3194625 | -8.2415 | 8.02E-29 | 6.92E-30 |
| osa-miR814a | 0 | 151.3194625 | -8.2415 | 8.02E-29 | 6.92E-30 |
| osa-miR817 | 0 | 151.3194625 | -8.2415 | 8.02E-29 | 6.92E-30 |
| osa-miR11336-5p | 132.2322263 | 504.3982082 | -1.9315 | 1.99E-25 | 1.69E-26 |
| osa-miR11339-3p | 264.4644525 | 756.5973123 | -1.5165 | 7.78E-21 | 6.19E-22 |
| novel_139 | 0 | 100.8796416 | -7.6565 | 1.50E-20 | 1.07E-21 |
| osa-miR1319a | 0 | 100.8796416 | -7.6565 | 1.50E-20 | 1.07E-21 |
| osa-miR1428e-3p | 0 | 100.8796416 | -7.6565 | 1.50E-20 | 1.07E-21 |
| osa-miR1429-5p | 0 | 100.8796416 | -7.6565 | 1.50E-20 | 1.07E-21 |
| osa-miR159a.2 | 0 | 100.8796416 | -7.6565 | 1.50E-20 | 1.07E-21 |
| osa-miR166a-5p | 0 | 100.8796416 | -7.6565 | 1.50E-20 | 1.07E-21 |
| osa-miR1846a-5p | 0 | 100.8796416 | -7.6565 | 1.50E-20 | 1.07E-21 |
| osa-miR1861g | 0 | 100.8796416 | -7.6565 | 1.50E-20 | 1.07E-21 |
| osa-miR1874-3p | 0 | 100.8796416 | -7.6565 | 1.50E-20 | 1.07E-21 |
| osa-miR1879 | 0 | 100.8796416 | -7.6565 | 1.50E-20 | 1.07E-21 |
| osa-miR2120 | 0 | 100.8796416 | -7.6565 | 1.50E-20 | 1.07E-21 |
| osa-miR444d.3 | 0 | 100.8796416 | -7.6565 | 1.50E-20 | 1.07E-21 |
| osa-miR5151 | 0 | 100.8796416 | -7.6565 | 1.50E-20 | 1.07E-21 |
| osa-miR812f | 0 | 100.8796416 | -7.6565 | 1.50E-20 | 1.07E-21 |
| osa-miR812g | 0 | 100.8796416 | -7.6565 | 1.50E-20 | 1.07E-21 |
| novel_44 | 132.2322263 | 50.43982082 | 1.3904 | 1.77E-19 | 1.22E-20 |
| osa-miR5072 | 132.2322263 | 50.43982082 | 1.3904 | 1.77E-19 | 1.22E-20 |
| osa-miR812p | 132.2322263 | 50.43982082 | 1.3904 | 1.77E-19 | 1.22E-20 |
| osa-miR169i-3p | 132.2322263 | 453.9583874 | -1.7795 | 2.96E-19 | 2.02E-20 |
| osa-miR169a | 132.2322263 | 403.5185665 | -1.6096 | 1.09E-13 | 7.05E-15 |
| osa-miR1878 | 396.6966788 | 907.9167747 | -1.1945 | 4.10E-12 | 2.61E-13 |
| osa-miR11338-5p | 0 | 50.43982082 | -6.6565 | 2.04E-11 | 1.10E-12 |
| osa-miR1429-3p | 0 | 50.43982082 | -6.6565 | 2.04E-11 | 1.10E-12 |
| osa-miR164c | 0 | 50.43982082 | -6.6565 | 2.04E-11 | 1.10E-12 |
| osa-miR171c-5p | 0 | 50.43982082 | -6.6565 | 2.04E-11 | 1.10E-12 |
| osa-miR172d-5p | 0 | 50.43982082 | -6.6565 | 2.04E-11 | 1.10E-12 |
| osa-miR1857-3p | 0 | 50.43982082 | -6.6565 | 2.04E-11 | 1.10E-12 |
| osa-miR1859 | 0 | 50.43982082 | -6.6565 | 2.04E-11 | 1.10E-12 |
| osa-miR1862f | 0 | 50.43982082 | -6.6565 | 2.04E-11 | 1.10E-12 |
| osa-miR1863b | 0 | 50.43982082 | -6.6565 | 2.04E-11 | 1.10E-12 |
| osa-miR1874-5p | 0 | 50.43982082 | -6.6565 | 2.04E-11 | 1.10E-12 |
| osa-miR2863c | 0 | 50.43982082 | -6.6565 | 2.04E-11 | 1.10E-12 |
| osa-miR2872 | 0 | 50.43982082 | -6.6565 | 2.04E-11 | 1.10E-12 |
| osa-miR2879 | 0 | 50.43982082 | -6.6565 | 2.04E-11 | 1.10E-12 |
| osa-miR3980a-3p | 0 | 50.43982082 | -6.6565 | 2.04E-11 | 1.10E-12 |
| osa-miR437 | 0 | 50.43982082 | -6.6565 | 2.04E-11 | 1.10E-12 |
| osa-miR439a | 0 | 50.43982082 | -6.6565 | 2.04E-11 | 1.10E-12 |
| osa-miR5073 | 0 | 50.43982082 | -6.6565 | 2.04E-11 | 1.10E-12 |
| osa-miR5148a | 0 | 50.43982082 | -6.6565 | 2.04E-11 | 1.10E-12 |
| osa-miR5337b | 0 | 50.43982082 | -6.6565 | 2.04E-11 | 1.10E-12 |
| osa-miR5801c-3p | 0 | 50.43982082 | -6.6565 | 2.04E-11 | 1.10E-12 |
| osa-miR5802 | 0 | 50.43982082 | -6.6565 | 2.04E-11 | 1.10E-12 |
| osa-miR5814 | 0 | 50.43982082 | -6.6565 | 2.04E-11 | 1.10E-12 |
| osa-miR6251 | 0 | 50.43982082 | -6.6565 | 2.04E-11 | 1.10E-12 |
| osa-miR7693-5p | 0 | 50.43982082 | -6.6565 | 2.04E-11 | 1.10E-12 |
| osa-miR7694-3p | 0 | 50.43982082 | -6.6565 | 2.04E-11 | 1.10E-12 |
| osa-miR810b.2 | 0 | 50.43982082 | -6.6565 | 2.04E-11 | 1.10E-12 |
| osa-miR812a | 0 | 50.43982082 | -6.6565 | 2.04E-11 | 1.10E-12 |
| osa-miR812n-3p | 0 | 50.43982082 | -6.6565 | 2.04E-11 | 1.10E-12 |
| osa-miR812v | 0 | 50.43982082 | -6.6565 | 2.04E-11 | 1.10E-12 |
| novel_45 | 132.2322263 | 353.0787457 | -1.4169 | 7.43E-09 | 3.92E-10 |
| osa-miR167d-3p | 132.2322263 | 353.0787457 | -1.4169 | 7.43E-09 | 3.92E-10 |
| osa-miR5504 | 132.2322263 | 353.0787457 | -1.4169 | 7.43E-09 | 3.92E-10 |
| osa-miR5150-3p | 264.4644525 | 554.838029 | -1.069 | 1.56E-05 | 7.84E-07 |
| novel_85 | 132.2322263 | 302.6389249 | -1.1945 | 6.27E-05 | 3.10E-06 |
| osa-miR396c-3p | 132.2322263 | 302.6389249 | -1.1945 | 6.27E-05 | 3.10E-06 |
| osa-miR5149 | 132.2322263 | 302.6389249 | -1.1945 | 6.27E-05 | 3.10E-06 |

**Table S4. List of the primers used in qPCR for miRNA and target gene expression analysis.**

| **Genes** | **Forward primer (5’-3’)** | **Reverse primer (5’-3’)** |
| --- | --- | --- |
| osa-miR530-5p | GGCGCTGCATTTGCACCTG |  |
| osa-miR6246 | GCTTGGGGATTTCCTGCCG |  |
| osa-miR812s | GAAGACGGACAATCAAACG |  |
| novel_16 | CCTGAAGCTGCCAGCATGA |  |
| novel_52 | GCCGCCGTTTTTGGAACAT |  |
| osa-miR3980a-5p | CCGCAATCGACGGCCTCAG |  |
| osa-miR399i | GGCCTGCCAAAGGAGAGCT |  |
| osa-miR156l-5p | CGGGCGACAGAAGAGAGTG |  |
| osa-miR164e | CCGCTGGAGAAGCAGGGCA |  |
| novel_139 | GGTTTCTCGATCTAACGGT |  |
| osa-miR2118g | CGGTTCCTAATGCCTCCCA |  |
| osa-miR435 | GCCGCTTATCCGGTATTGG |  |
| osa-miR156k | GGGCTGACAGAAGAGAGAG |  |
| osa-miR166g-5p | CCCGAATGGAGGCTGATCC |  |
| osa-miR1882e-3p | CGGAAATGATCTTGGACGT |  |
| osa-miR2871a-3p | GCGGCCGTATTTTAGTTTCT |  |
| osa-miR172a | CGCGAGAATCTTGATGATG |  |
| novel_126 | CAGTCCTCACGTGGGCATA |  |
| osa-miR167e-3p | GGCCAGATCATGTTGCAGC |  |
| osa-miR1879 | CGTGTTTGGTTTAGGGATG |  |
| U6-F | TACAGATAAGATTAGCATGGCCCC |  |
| U6-R | GGACCATTTCTCGATTTGTACGTG |  |
| Universal-R |  | GTGCAGGGTCCGAGGTATTC |
| LOC_Os03g55784 | TGCAGCACTATTCTCTGCCT | GCCTCAGCCAACTGGAAAGA |
| LOC_Os04g01570 | CGTCACAGTCCACCAGGC | GGCATGAGGCTCCGCTC |
| LOC_Os01g69830 | GCAGGTTTCATTTGCTCGGG | CCGTGGTGATTGGCGAAAAG |
| LOC_Os02g36880 | CATCACGCACTACCTCCTCC | AGTACCACTCCTTCTCCCCC |
| LOC_Os08g42700 | CTCATCACCAACGACGGACA | CAGGCGTGGCATTCAAACTC |
| LOC_Os07g41730 | GAACTGTGTTGTTCGCTCCC | AACCTCATGTGTGAAGGGGG |
| LOC_Os10g13810 | GAAGGTAGTCGTGTGGTGGG | TGACCTCTGTTCTTGGCGTC |
| LOC_Os05g03040 | GCAGCTCAAAGTACAGGGGT | CATAAGCCCTTGCAGCCTCT |
| LOC_Os06g46770 (Ubiquitin) | CCAGTAAGTCCTCAGCCATGGAG | GGACACAATGATTAGGGATC |

**Table S5. List of the predicted miRNA targets having a putative defense modulatory role in rice. The targets that showed an expectancy value ≤ 3, while their corresponding miRNA had a fold change (log_2_) of ≥ 2 and were not common in between IR56-IR and IR56-TN1 selected for the analysis.**

| **miRNA** | **Target** | **Putative function** |
| --- | --- | --- |
| **IR56-IR vs IR56-CK** | | |
| osa-miR812s | LOC_Os04g01570  LOC_Os07g39830 | invertase/pectin methylesterase inhibitor family protein  F-box domain, cyclin-like domain containing protein |
| osa-miR530-5p | LOC_Os03g55784  LOC_Os02g14990 | Allene oxide synthase.  zinc finger, C3HC4 type domain containing protein |
| osa-miR6246 | LOC_Os03g11890  LOC_Os04g58020 | Conserved hypothetical protein (OBE4)  MYB_Al protein |
| osa-miR166e-3p | LOC_Os03g43930.2  LOC_Os01g13000 | HD-Zip protein TF  ethylene-responsive protein related |
| osa-miR1870-3p | Os07g0648233  LOC_Os03g24860 | Armadillo-type fold domain containing protein.  transporter family protein |
| osa-miR393b-3p | LOC_Os01g07590  LOC_Os05g03550 | universal stress protein domain containing protein  MYB family transcription factor |
| osa-miR166a-5p | LOC_Os03g09140  LOC_Os12g14610 | ras-related protein  cysteine-rich receptor-like protein kinase 32 precursor |
| osa-miR5159 |  |  |
| novel_16 | LOC_Os06g03830.1  LOC_Os05g01780 | SDR family protein.  STE kinase Wnk.1 |
| novel_52 | LOC_Os04g23700 | lectin protein kinase family protein |
| novel_128 | LOC_Os06g47650.1  LOC_Os03g20420 | Protein kinase, core domain containing protein  alpha-N-arabinofuranosidase A |
| novel_47 | LOC_Os02g49326.1  LOC_Os01g74330 | Ribulose-1,5-bisphosphate carboxylase/oxygenase small subunit N-methyltransferase I  pectinacetylesterase domain containing protein |
| osa-miR7694-3p | LOC_Os12g14699  LOC_Os03g50850 | protein kinase domain containing protein  C2H2 zinc finger protein |
| osa-miR5337a | LOC_Os02g12910  LOC_Os02g12910 | receptor-like protein kinase 5 precursor  receptor-like protein kinase 5 precursor |
| osa-miR1852 | LOC_Os01g61620  LOC_Os02g07840 | protein kinase family protein  bZIP transcription factor domain containing protein |
| osa-miR1857-5p | LOC_Os12g01180  LOC_Os02g30900 | WRKY57  protein kinase domain containing protein |
| osa-miR2864.1 | LOC_Os10g07534 | disease resistance protein RPM1 |
| osa-miR3980a-5p | LOC_Os08g01390.1  LOC_Os01g72260 | Chaperonin Cpn60/TCP-1 family protein.  cytochrome P450 |
| osa-miR399i | LOC_Os04g55230 | Tetratricopeptide repeat (TPR) domain containing protein |
| osa-miR156l-5p | LOC_Os01g69830 | OsSPL2 - SBP-box gene family member |
| osa-miR164e | LOC_Os02g36880 | No apical meristem protein |
| osa-miR5539a | LOC_Os05g24970 | LSM domain containing protein |
| novel_139 | LOC_Os11g03310 | NAM protein putative |
| **IR56-TN1 vs IR56-CK** | | |
| osa-miR2118g | LOC_Os08g42700  LOC_Os01g20720 | NB-ARC domain containing protein  CC-NBS-LRR, putative |
| osa-miR435 | LOC_Os07g41730  LOC_Os02g05930 | Alpha/beta hydrolase fold-1 domain containing protein  phytosulfokine receptor precursor |
| osa-miR156k | LOC_Os01g69830  LOC_Os11g30370 | OsSPL2 - SBP-box gene  OsSPL19 - SBP-box gene |
| osa-miR166g-5p | LOC_Os01g64730 | bZIP transcription factor domain containing protein |
| osa-miR1882e-3p | LOC_Os02g02860  LOC_Os03g62510 | glutamyl-tRNA synthetase, putative  amine oxidase, flavin-containing, domain containing protein |
| osa-miR166d-5p | LOC_Os10g26730 | exonuclease, putative, expressed |
| osa-miR2871a-3p | LOC_Os10g13810 | glycosyltransferase family 43 protein |
| osa-miR172a | LOC_Os05g03040 | Starch biosynthesis- AP2/EREBP family transcription factor |
| osa-miR167e-3p | LOC_Os03g17120  LOC_Os03g49140 | arginine biosynthesis bifunctional protein argJ 1  ABC1 protein At2g40090 precursor |
| osa-miR166k-5p | LOC_Os10g23090  LOC_Os03g61950 | homeobox associated leucine zipper  kelch motif family protein |
| osa-miR166a-5p | LOC_Os11g40430  LOC_Os12g14699  LOC_Os03g59390 | wall-associated receptor kinase-like 2 precursor  protein kinase domain containing protein  CAMK_CAMK_like.24, calcium/calmodulin depedent protein kinases |
| osa-miR1879 | LOC_Os12g19090  LOC_Os03g63060  LOC_Os11g35120 | metalloprotease ATP23  P21-Rho-binding domain containing protein  OsWAK116 - OsWAK receptor-like cytoplasmic kinase OsWAK-RLCK |
| osa-miR444d.3 | LOC_Os02g49840 | OsMADS57 - MADS-box family gene with MIKCc type-box |
| osa-miR1859 | LOC_Os01g12860  LOC_Os01g25810  LOC_Os01g23530 | MYB family transcription factor  powdery mildew resistance protein PM3A  terpene synthase |
| osa-miR1423-5p | LOC_Os02g46760 | STE_MEK_ste7_MAP2K.4 - STE kinases homolog |
| osa-miR810b.2 | LOC_Os11g02840 | protein kinase, putative |
| osa-miR1850.1 | LOC_Os04g47410 | DHHC zinc finger domain containing protein |
| osa-miR2880 | LOC_Os11g29720 | cytochrome P450 |
| osa-miR444d.3 | LOC_Os02g49840  LOC_Os10g27170 | OsMADS57 - MADS-box family gene with MIKCc type-box  calmodulin-binding protein |
| novel_99 | LOC_Os11g34880 | NB-ARC domain containing protein |
| novel_126 | LOC_Os12g35340 | OsGrx_C11 - glutaredoxin subgroup III, expressed |
